# Weaning-associated feed deprivation stress causes microbiota disruptions in a novel mucin-containing *in vitro* model of the piglet colon (MPigut-IVM)

**DOI:** 10.1186/s40104-021-00584-0

**Published:** 2021-06-02

**Authors:** Raphaële Gresse, Frédérique Chaucheyras-Durand, Sylvain Denis, Martin Beaumont, Tom Van de Wiele, Evelyne Forano, Stéphanie Blanquet-Diot

**Affiliations:** 1 Université Clermont Auvergne, INRAE, UMR 454 MEDIS, F-63000 Clermont-Ferrand, France; 2 Lallemand SAS, F-31702 Blagnac, Cedex France; 3 GenPhySE, Université de Toulouse, INRAE, ENVT, F-31326 Castanet-Tolosan, France; 4 Ghent University, Center for Microbial Ecology and Technology, B-9000 Ghent, Belgium

**Keywords:** Colon, Dysbiosis, *In vitro* gut model, Microbiota, Mucin, Piglet, Weaning

## Abstract

**Background:**

Risk factors for the etiology of post-weaning diarrhea, a major problem in swine industry associated with enormous economic losses, remain to be fully elucidated. In concordance with the ethical concerns raised by animal experiments, we developed a new *in vitro* model of the weaning piglet colon (MPigut-IVM) including a mucin bead compartment to reproduce the mucus surface from the gut to which gut microbes can adhere.

**Results:**

Our results indicated that the MPigut-IVM is able to establish a representative piglet archaeal and bacterial colon microbiota in terms of taxonomic composition and function. The MPigut-IVM was consequently used to investigate the potential effects of feed deprivation, a common consequence of weaning in piglets, on the microbiota. The lack of nutrients in the MPigut-IVM led to an increased abundance of Prevotellaceae and *Escherichia-Shigella* and a decrease in Bacteroidiaceae and confirms previous *in vivo* findings*.* On top of a strong increase in redox potential, the feed deprivation stress induced modifications of microbial metabolite production such as a decrease in acetate and an increase in proportional valerate, isovalerate and isobutyrate production.

**Conclusions:**

The MPigut-IVM is able to simulate luminal and mucosal piglet microbiota and represent an innovative tool for comparative studies to investigate the impact of weaning stressors on piglet microbiota. Besides, weaning-associated feed deprivation in piglets provokes disruptions of MPigut-IVM microbiota composition and functionality and could be implicated in the onset of post-weaning dysbiosis in piglets.

**Supplementary Information:**

The online version contains supplementary material available at 10.1186/s40104-021-00584-0.

## Background

In modern swine industry, piglet weaning occurring mostly between 3 to 4 weeks of age is the most critical event in pig life due to sudden social, environmental and dietary stresses [1]. Weaning is frequently associated with changes in diet composition but also considerably impacts the behavior of piglets resulting in vocalizations, fighting, and generally, weaning anorexia [1–3]. Social, dietary and environmental stresses are also associated with numbers of physiological changes including modifications in piglet intestinal microbiota [4]. Piglet intestinal microbiota normally varies in population density and diversity across the successive gut organs and between the lumen and the mucosa but encounters a real microbial shift consequently to the weaning transition [5, 6]. If weaning-associated disruptions of microbiota functional capacities are poorly described yet, several studies reported changes in microbiota composition such as an increase or decrease in bacterial richness and diversity, sharp increases in Prevotellaceae, increases in Clostridiaceae*,* reductions in *Lactobacillus, Bacteroides* and complete disappearance of *Fusobacterium* or modifications of the Firmicutes*/*Bacteroidetes ratio in feces or intestinal contents [6–11].

Among the numerous weaning stressors, post-weaning-associated fasting periods affect growth performance and body weight [12, 13]. Piglets approximately lose about 100–250 g body weight the first day after weaning which directly affects the total days to market [13]. Reduced feed intake after weaning is variable from 24 h to 4 d depending of individuals [14] and has been reported to be the most important factor compromising intestinal barrier function ahead of dietary change [15]. The lack of nutrients in the intestine of piglets may contribute to intestinal inflammation and changes in intestinal morphology such as reduced villus height and increased intestinal permeability which facilitate the crossing of the mucus layer and the intestinal epithelial barrier by toxins and bacteria [3, 12, 16]. This mucus layer is a permeable gel overlying intestinal epithelial cells, separating them from gut luminal content, including commensal bacteria and invading pathogens [17]. The mucus-associated microbiota, also referred to as mucobiota [18], uses specific adhesins to bind to the polysaccharides composing mucins [17]. The mucobiome is thought to be an important regulator of host health. Such as in the lumen, a disproportion of mucus-associated microbial communities could impact the balance between gut commensals and thus lead to the development of a disrupted microbiota and impaired microbial functions [17], also called dysbiosis. Regarding its numerous reported effects, the weaning-associated feed deprivation period could play a role in the etiology of post-weaning intestinal dysbiosis due to the depletion of nutrients and the increased degradation of mucins and lead to a higher risk of developing enteric infections also called post-weaning diarrhea. To fight against post-weaning diarrhea, current strategies generally involve preventive and curative treatments with antibiotics which raise serious public health concerns due to the increasing antibiotic resistance prevalence [4, 19]. Thus, there is a real need to find non-antibiotic solutions such as dietary compounds, prebiotics or probiotics, and restore gut microbial balance at weaning.

Even though *in vivo* experiments remain the gold standard for evaluating the effects of non-pharmacological compounds on gut microbiota, pig *in vitro* digestion models constitute first-choice alternatives for ethical, technical, cost, and regulatory reasons. Continuous *in vitro* fermentation models can closely mimic pig colon physiology by simulating pH of colonic environment, consistent transit time, anaerobic habitat and supply of a nutrient medium close to the composition of ileal or caecal effluents, while maintaining a functional microbiota [20–24]. Few semi-continuous or continuous *in vitro* models of the pig colon have been developed but the Pigut-IVM (Piglet Gut *In vitro* Model) and the BABY-SPIME (Baby Simulator of Pig Intestinal Microbial Ecosystem) are the only developed models aiming to reproduce the specific conditions encountered in the colon lumen of piglets [22, 24]. Up to date, none of these models is able to mimic the gut mucosal surface. Incorporating mucin in fermentation models would make a surface available for microbial adhesion in order to get a more complete picture of the colonic ecosystem. The aim of this study was thus to develop, based on *in vivo* data, a continuous *in vitro* fermentation model including a mucin bead compartment in order to reproduce at best the colonic luminal and mucus microenvironments of 4-week old piglets. Once developed, this *in vitro* model was applied to evaluate the consequences of 48 h feed deprivation on piglet microbiota composition and functionality, which was used to simulate a weaning feed deprivation stress.

## Materials and methods

### Samples collection and treatments

All animals were housed in a conventional pig farm located in the Haute-Loire area of the Auvergne-Rhône-Alpes region in France. Piglets remained with their mother and siblings during the suckling period. In addition to sow milk, piglets received water and pre-weaning diet *ad libitum* 1 weeks after birth. None of the piglets had signs of enteric or metabolic disturbances. The animals did not receive any antibiotic in the 27 d prior to sample collection day. As freezing process showed to affect bacterial abundances in pig feces [25], fecal samples from suckling 4-week old healthy male piglets belonging to different litters and destined to fermentation runs (Landrace × Large White) were collected using sterile bottles and immediately stored at 4 °C under anaerobiosis conditions using GENbag anaer gas pack systems (Biomerieux, Marcy L’Etoile, France) during the 1.5 h transport to laboratory. In order to make a reliable comparison between our *in vitro* colonic model with *in vivo* colonic samples, luminal and mucosal proximal colon samples were collected from 5 healthy male piglets after slaughter on site. For collection of mucosal scrapings, the proximal colon was rinsed with sterile PBS to remove any digesta and its surface was scrapped using a sterile microscope glass slide. The experimental procedures were followed in accordance with the guidelines established by the European Community Council under the Directive 2010/63/EU. The experiments were exempted from ethic evaluations because all animals were commercially raised and slaughtered on site under the supervision of the local veterinary. Proximal colon mucosal and luminal samples were collected from the study of Gresse et al. where procedures are further described [26].

### Description of the MPigut-IVM parameters

**Fig. 1:**
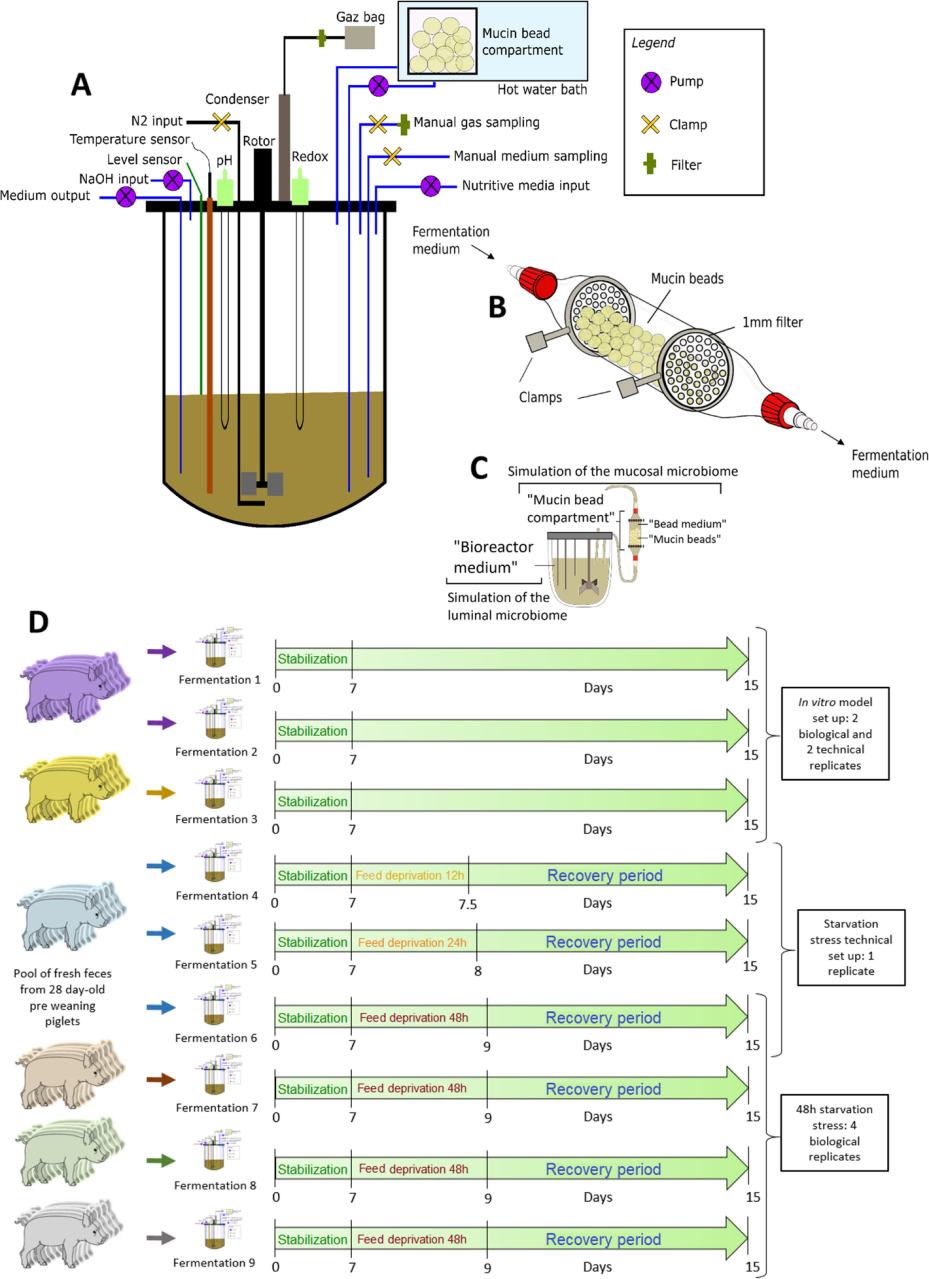
Description of the new mucin implemented *in vitro* of the piglet colon (MPigut-IVM). **a** Schematic view of the MPigut-IVM. **b** Schematic representation of the mucin bead compartment of the MPigut-IVM. **c** Denomination of the MPigut-IVM samples throughout this publication. **d** Experimental design of the fermentation experiments performed using the MPigut-IVM

Five hundred mL MiniBio bioreactors (Applikon Biotechnology, Delft, The Netherlands) equipped with stirrers, ports and probes (Fig. 1a) were used under continuous conditions to simulate the colonic environment based on *in vivo* data collected previously in the same commercial pig farm [26]. For the inoculation of one bioreactor, feces from 6 to 8 piglets housed in the same stable, corresponding to 10 g, were pooled and mixed under anaerobic conditions with 30 mmol/L sterile sodium phosphate buffer (pH 6.0) to reach a total volume of 50 mL. The fecal inoculum was filtered through a 500-μm inox sieve. Fifty mL of the fecal suspension were added per bioreactor to 150 mL of nutritive medium (see below) while flushing with O_2_-free N_2_ gas, in order to ensure anaerobic conditions at the beginning of fermentation. Afterwards, during the fermentation course, the anaerobic conditions were maintained exclusively through the activity of the resident microbiota and by ensuring the airtightness of the system. The temperature of the fermentation was set up to 39 °C corresponding to the physiological temperature of piglet colon [22], controlled using a specific probe, and maintained inside the main tank using an incorporated panel heater and inside the mucin bead compartment using a hot water bath. pH was recorded every 300 s in the bioreactor medium using a pH sensor (Mettler Toledo, Viroflay, France) and adjusted to a value of 6.0 corresponding to 28 days-old piglet colon [26] with an automatic addition of 2 mol/L NaOH. Redox potential was constantly measured in the bioreactor medium using a redox sensor (Mettler Toledo, Viroflay, France). The fermentation medium was stirred at a constant speed of 300 r/min during the total duration of the experiment. The nutritive medium was continuously introduced into the bioreactors using a peristaltic pump at an average speed of 0.17 mL/min. The volume of bioreactors was monitored using a level sensor and maintained at a constant value of 200 mL by automatic withdrawal of the fermentation medium. The constant arrival speed of the nutritive medium inside the bioreactors and the regular withdraw of the fermentation medium ensured a retention time of 19.6 h to mimic the colonic retention time of 4-week old piglets [27]. A nutritive medium formula containing various sources of carbohydrates, proteins, minerals and vitamins was set up using both the method used by McFarlane et al. [28]*,* Fleury et al. [21] and on the basis of ingredients generally found in piglet feed at weaning, digestibility indices of ingredients,and the nutrients or growth factors necessary to maintain a viable microbiota in bioreactors (Supplementary Table 1). Calculations were based on estimation that piglets of this age would have a feed intake of 500 g per day. For example, digestibility indices of 95% for wheat starch, 94% for soy meal and 95% for whey powder were used to calculate the final concentration of these ingredients in the nutritive medium mimicking ileal chyme considering a basal diet for piglets containing 30–60% of wheat starch, 5 to 10% of soy meal and 10–40% of whey powder. Example of calculations for soy meal: if the basal diet contains 8% of soy meal we can consider that 40 g from the 500 g ingested by day are soy meal. From this 40 g of soy meal ingested, only 6% will get to the colon so 2.4 g per day. If the nutritive medium flow to the speed of 0.17 mL/min × 1,440 min/d = 244 mL are consumed per day. Therefore our medium should contains, (2.4×1,000)/244 = 9.84 rounded to 10 g/L of soy proteins. Anaerobic conditions and gas composition were checked every day by analyzing O_2_, CO_2_, CH_4_ and H_2_ produced during the fermentation process in the atmospheric phase of the bioreactors using a HP 6890 Gas Chromatograph (Agilent Technologies, Santa Clara, USA) coupled with a TCD detector (Agilent Technologies, Santa Clara, USA) and two series columns, Molecular Sieve 5A and Porapack Q (Aligent Technologies, Santa Clara, USA).

### Mucin bead production and compartment

Mucin from porcine stomach type II (Sigma-Aldrich, Saint-Louis, Missouri, USA) and sodium alginate (Sigma-Aldrich, Saint-Louis, Missouri, USA) were diluted in sterile distilled water, at a concentration of 5% and 2%, respectively. Sterile sodium bicarbonate 1 mol/L was used to adjust the pH at 6.0. To produce mucin beads of 4 mm of diameter, the mucin solution was dropped using a peristaltic pump and a 2.06 mm × 0.85 mm Tygon R3607 pipe (Saint Gobain, Courbevoie, France) at an approximate height of 40 cm into a 0.2 mol/L solution of sterile CaCl_2_ under agitation. The mucin beads occupied the mucin bead compartment volume of 50 mL out of the 200 mL of the total MPigut-IVM volume. At the beginning of fermentations, 350 ± 20 mucin beads were introduced in specific glass compartments allowing the circulation of the fermentation medium on its entire length and ensuring the contact of the resident microbiota with the mucin beads. The bead volume/liquid volume ratio was equal to 7.85 mL. The pump speed of the reactor effluent over the bead compartment was 5 mL/min and was technically chosen to avoid blockage inside the pipes. The temperature of the mucin bead compartment was maintained at 39 °C using a hot water bath. Mucin beads were maintained in the compartments using Teflon plastic filters possessing pores of 1 mm of diameter (Verre Labo Mula, Vaulx-en-Velin, France). One mucin bead compartment was connected to one mini-bioreactor medium using pipes and one peristaltic pump. The specific design of the mucin bead compartment is illustrated in Fig. 1b and an overview of the whole system is shown in Fig. 1c. During the fermentation process, the mucin beads were totally replaced every 48 h to ensure a continuous availability of mucin adherent surfaces. During the time of bead replacement, the medium of the bead compartment was kept under CO_2_ flushing to avoid oxygen entrance.

### Experimental design of fermentations using the newly developed MPigut-IVM

#### Control fermentations

The aim of these fermentation runs was to ensure that a viable and functional resident microbiota, comparable to the microbiota found *in vivo* in the colon of piglets, was maintained inside the fermentation vessels and on the mucin beads for a period of 15 d. As a strong control, both technical and biological replicates were performed without any treatment and to determine the number of days necessary to allow microbial populations to stabilize (Fig. 1d). Control assays lasted 15 d as a compromise between a good stability of microbial communities and the focus on the first few days after weaning.

#### Application to the simulation of weaning stressors

The MPigut-IVM was then applied in a context of dietary stress, i.e. a feed deprivation period that piglets frequently encounter at weaning. To set up the most appropriate duration of the feed deprivation and evaluate its impact, the first experiment consisted in applying a feed deprivation period of 12, 24 and 48 h in 3 bioreactors inoculated with the same fecal pooled sample. To simulate feed deprivation, the flow of nutritive medium was interrupted during 12, 24 or 48 h 7 d after starting the fermentation. The 48 h feed deprivation experiment was in total repeated 4 times with 4 different fecal inocula and fermentations were numbered from 1 to 4 (Fig. 1d). To avoid air contamination during the feed deprivation period which could be due to a depression caused by the stop of fermentative activity inside the system, a bag was filled with carbon dioxide and connected to the bioreactor using a one way valve.

#### Sampling of the MPigut-IVM

Samples from bioreactor mediums also referring to the luminal microbiota were collected every day. As mucin beads and bead medium were collected every 48 h, no sampling was carried out during the feed deprivation periods but before (day 7) and after (day 9) (Fig. 1d). Samples from the bioreactor medium and bead medium were centrifuged at 4 °C, 10,000 × *g* and for 45 min. Pellets and supernatants were respectively stored at − 20 °C and − 80 °C until analysis. After collection, mucin beads were gently washed 3 times in sterile 1× PBS and stored at − 20 °C.

### Observation of the mucin beads by scanning electron microscopy (SEM)

To control that microorganisms were able to adhere to the bead surface during the fermentation process, some beads were sampled after a 48-h stay in the mucin bead compartment during a control assay. Beads were gently washed 3 times in sterile PBS 1×. Then, they were fixated by immersion at 4 °C overnight in a 0.2-mol/L sodium cacodylate (pH 7.4) buffer containing 2% glutaraldehyde. After fixation, mucin beads were dehydrated using successive 20 min incubations in ethanol 70%, 95% and 100%. A final dehydration step included 20 min incubation in a mix of ethanol 100% and hexamethyldisilazane. Samples were dried overnight under a chamber and stained with gold-palladium deposition. SEM images were taken with a JEOL 6060 Low vacuum microscope (Jeol Europe SAS, Croissy sur Seine, France) at the Centre Imagerie Cellulaire Santé (CICS) from Clermont-Auvergne University.

### DNA extraction from MPigut-IVM samples

Total DNA was extracted from all samples using the Quick-DNA Fecal/Soil Microbe Miniprep Kit (Zymo Research, Irvine, CA, USA) according to the manufacturer’s instructions. For mucin beads, defrost samples were homogenized and 250 μL of this mixture was used for DNA extraction. The pellet used for DNA extraction was weighted for each sample. The quality of the eluted DNA was assessed by agarose gel electrophoresis. Extracts were quantified using the Qubit dsDNA Broad Range Assay Kit (Invitrogen, Carlsbad, CA, USA) with a Qubit 2.0 Fluorometer (Invitrogen, Carlsbad, CA, USA). Samples were stored at − 20 °C prior to analysis.

### Standard curve assessments for quantitative PCR analyses

Conventional PCR for the amplification of the 16S ribosomal gene was carried out on genomic DNA from *Escherichia coli* (DSMZ N° 30083), *Bacteroides pyogenes* (DSMZ N°20611), *Prevotella stercorea* (DSMZ N°18206), *Lactobacillus mucosae* (DSMZ N°13345), *Faecalibacterium prausnitzii* (DSMZ N°17677) and *Dorea formicigenerans* (DSMZ N°3992) (DSMZ, Braunschweig, Germany) which were used as respective standards for the quantification of *Escherichia Shigella* and total bacteria, Bacteroidetes*, Prevotella,* Firmicutes, *Clostridium* cluster IV and *Clostridium* cluster XIVa groups. The reaction was performed using the universal 16S primers 8F 5′-ACTCCTACGGGAGGCAG-3′ and 1492R 5′-GTATTACCGCGGCTGCTG-3′ and the Platinum™ Taq DNA Polymerase kit (Invitrogen, Carlsbad, CA, USA). The PCR was carried out with a Bio-Rad iCycler thermal cycler (Bio-Rad, Hercules, CA, USA) under the following conditions: one cycle of 94 °C for 2 min; 30 cycles of (94 °C for 30 s, 52 °C for 30 s, and 72 °C for 90 s). The PCR products were purified using the QIAquick PCR Purification Kit (Qiagen, Venlo, The Netherlands) according to the manufacturer’s instructions and were subjected before and after purification to a 1% agarose gel electrophoresis containing ethidium bromide and visualized for being approximately equal to 1,484 bp using the ladder 500 bp Mol Ruler (Bio-Rad, Hercules, CA, USA). DNA concentration was measured with a Qubit dsDNA Broad Range Assay Kit (Invitrogen) using a Qubit 2.0 Fluorometer (Invitrogen, Carlsbad, CA, USA). The 16S rDNA gene copy number was calculated using the formula: copy number/μL = (C/X)×0.912.1012 with C: DNA concentration measured (ng/μL) and X: PCR fragment length (bp/copy) and diluted in 10-fold dilution series to be used as qPCR standards. Efficiency of the qPCR for each target varied between 95% and 105% with a slope from − 3.0 to − 3.4 and a regression coefficient above 0.95, which was in accordance with the MIQE guidelines [29].

### Quantification of bacterial groups and methanogenic archaea populations by qPCR

The list of primer pairs and their optimal conditions used for quantitative PCR are presented in Supplementary Table 2 [30–36]. Real-time PCR assays were performed on a Rotor-Gene Q (Qiagen, Venlo, The Netherlands) in 20 μL reactions with QuantiFast SYBR GREEN master mix (Qiagen, Venlo, The Netherlands) or Taqman Fast Advanced Master mix (Applied Biosystems, Foster City, California, USA) with the additions of each primer at their optimal concentration (Supplementary Table 2). The 16S rDNA genes were amplified using the following program: 2 min denaturation at 95 °C and 10 min denaturation at 95 °C; 40 and 45 cycles of 20 s at 95 °C and 60 s elongation and extension at the optimum annealing temperature and, when performing SYBR GREEN based assay, a melt curve step from 60 °C to 95 °C. After log transformation of the data, a mixed-model one-way ANOVA (lmer and ANOVA functions) with time point (days of fermentation) as a fixed effect and fermentation experiment as a random effect was used to compare the number of 16S gene copy per g of samples between days of fermentation using the R packages lme4 and car [31–36].

### MiSeq 16S rDNA sequencing and bioinformatic analysis

The DNA concentration of all samples was measured using the Qubit dsDNA High Sensivity Assay Kit (Invitrogen, Carlsbad, CA, USA) with a Qubit 2.0 Fluorometer (Invitrogen, Carlsbad, CA, USA) and diluted to 2 ng/μL prior to PCR amplification. The Bacterial V3-V4 region of 16S rDNA and the Archaeal 16S rDNA were respectively amplified with primers 357F 5′-CCTACGGGNGGCWGCAG-3′ [32] and 805R 5′-GACTACHVGGGTATCTAATCC-3′ [37] and primers 349F 5′-GYGCASCAGKCGMGAAW-3′ and 806R 5′-GGACTACVSGGGTATCTAAT-3′ [34]. Amplicons were generated using a Fluidigm Access Array followed by high-throughput sequencing on an Illumina MiSeq system (Illumina, San Diego, CA, USA) performed at the Carver Biotechnology Center of the University of Illinois (Urbana, IL, USA). The demultiplexed paired end Illumina sequence reads in the FastQ format were uploaded into the Galaxy instance (v.2.3.0) of the Genotoul bioinformatics platform (http://sigenae-workbench.toulouse.inra.fr) to be used in the FROGS (Find Rapidly OTU with Galaxy Solution) pipeline [38]. During the FROGS pre-process, sequences were depleted of barcode and the sequences with a non-appropriate length or containing ambiguous bases were removed. Next, reads were clustered into de novo operational taxonomic units (OTUs) using SWARM algorithm [39] with, at first, a denoising step to build very fine cluster using the minimal distance equal to 1 and, secondly, with an aggregation distance equal to 3. Chimeras were then detected and removed with VSEARCH [40]. Additionally, filters were applied to the OTUs in order to remove singletons [41, 42]. The OTUs selected were taxonomically assigned using the Silva release 132 reference database [43].

### Statistical analysis of sequencing data

Statistical analysis was processed using the RStudio software version 1.0 (with R software version 3.5.1, R Development Core Team, http://www.R-project.org). OTU structure and composition analyses were performed using the phyloseq R package version 1.30.0 [44]. Visualization of data was performed using the ggplot2 R package version 3.2.1. Prior to alpha and beta diversity calculations, rarefaction using the transform counts methods was applied to the dataset. The following alpha diversity indices were calculated: Inverse Simpson index, Chao 1 index, number of observed OTU phylogenetic diversity (PD) and Shannon index. Statistical differences in Bray Curtis distance between the mucin beads and the bioreactor medium were tested using a multi-analysis of variance (MANOVA) performed with ADONIS using the vegan R package with 9999 permutations and represented by principal coordinate analysis (PCoA) plots. The relative abundances of bacterial groups were log transformed prior to univariate statistical analyses. All univariate statistical analyses were performed using linear mixed-models (lme4 package version 1.1.21) with time point (days of fermentation) as a fixed effect and fermentation experiment as a random effect. Analysis of variance tables were calculated with the car package (version 3.0.6). The means of each group were compared pairwise with the lsmeans package (version 2.30-0) with the Tukey correction. Statistical comparisons of samples from the stabilization and recovery phases of the fermentation 6, 7, 8 and 9 were performed using the Wald test of the DESeq2 R package version 1.26.0 at the genus level. In all statistical analyses, only *P-*values below 0.05 were considered as significant.

### Quantification of short chain fatty acids (SCFAs) by gas chromatography

The SCFAs were quantified in the bioreactor medium and bead medium by gas chromatography in order to determine concentrations of acetate, propionate, isobutyrate, butyrate, caproate, isovalerate and valerate. Eight hundred μL of fermentation supernatants were mixed with 500 μL of 0.4% (w:v) crotonic acid and 2% (w:v) metaphosphoric acid solutions. This mixture was centrifuged again and the supernatant obtained was injected in a PerkinElmer Clarus 580 gas chromatograph (Waltham, Massachusetts, USA) for quantification of SCFAs. A mixed-model one-way ANOVA (lmer and ANOVA functions) with time point (days of fermentation) as a fixed effect and fermentation experiment as a random effect was used to compare the concentration of the main SCFAs between days of fermentation using the R packages lme4 and car.

### Metabolite analysis by ^1^H nuclear magnetic resonance (NMR)

Supernatants of the bioreactor medium, bead medium of the fermentations #6, 7, 8 and 9 were used for metabolomic profiling using NMR spectroscopy. Fermentation supernatants were centrifuged twice (18,000×*g*, 4 °C, 10 min) to remove particles. Fifty μL were mixed with 600 μL NMR buffer composed of sodium phosphate 0.2 mol/L, pH 7.4, trimethylsilylpropanoic acid 1 mmol/L, 80% deuterated water, and 20% water. The samples were homogenized, centrifuged and 600 μL were transferred to 5-mm NMR tubes. All NMR spectra were obtained with an Avance III HD NMR spectrometer operating at 600.13 MHz for ^1^H resonance frequency using a 5 mm inverse detection CryoProbe (Bruker Biospin, Rheinstetten, Germany) in the MetaboHUB-MetaToul-AXIOM metabolomics platform (Toulouse, France). ^1^H NMR spectra were acquired at 300 K using the Carr-Purcell-Meiboom-Gill spin-echo pulse sequence with presaturation. Pre-processing of the spectra (group delay correction, solvent suppression, apodization with a line broadening of 0.3 Hz, Fourier transform, zero order phase correction, shift referencing on TSP, baseline correction, setting of negative values to zero) was performed in the galaxy tool Workflow4Metabolomics following guidelines [45]. After water region (4.5–5.1 ppm) exclusion, spectra (0.5–9 ppm) were bucketed (0.01 ppm bucket width) and normalized by total area in Workflow4Metabolomics. Representative samples were characterized by 2D NMR experiments (^1^H-^1^H COSY and ^13^C-^1^H HSQC). For metabolite identification, 1D and 2D NMR spectra of pure compounds prepared in the same buffer and acquired with the same spectrometer were overlayed with samples spectra. Annotated representative spectra are presented in Supplemental Figure 1. For each identified metabolite, buckets non-overlapping with other metabolites were selected for the quantification (Supplemental Table 4).

Statistical analysis for NMR metabolomics were performed using the R software (version 3.5.1). Partial-least square discriminant analysis (PLS-DA) were performed with mixOmics package [46]. Metabolites relative concentrations was used as variable matrix (X). For PLS-DA, time points (days of fermentation) were used as predictors (Y) and time-repeated measurement were considered by using a multilevel approach. Univariate statistical analysis was also performed on each metabolite relative concentration with the R packages lme4 and car. A mixed-model one-way ANOVA (lmer and ANOVA functions) with time point (days of fermentation) as a fixed effect and fermentation experiment as a random effect was used. A post-hoc test was used to compare the mean relative concentrations with Tukey correction. *P*-values were corrected for multiple testing (false discovery rate). Spearman correlation was used to correlate the *in vitro* metabolome with the 20 most abundant families of the MPigut-IVM using the cor function of the R software. *P*s were corrected for multiple testing using the FDR method and the package Hsmic on the R sofltware.

## Results

### Set-up and validation of the MPigut-IVM (fermentations #1, 2 and 3)

#### A fermentative microbial activity is successfully maintained in the MPigut-IVM

Redox potential was recorded every 300 s during the 15 d of the fermentation process (fermentation #1, 2, 3). During the first 24 h of fermentation, the redox potential quickly decreased from the starting value of approximately –150 mV to the average value of − 261 ± 7 mV indicative of strong anaerobic conditions.

**Fig. 2:**
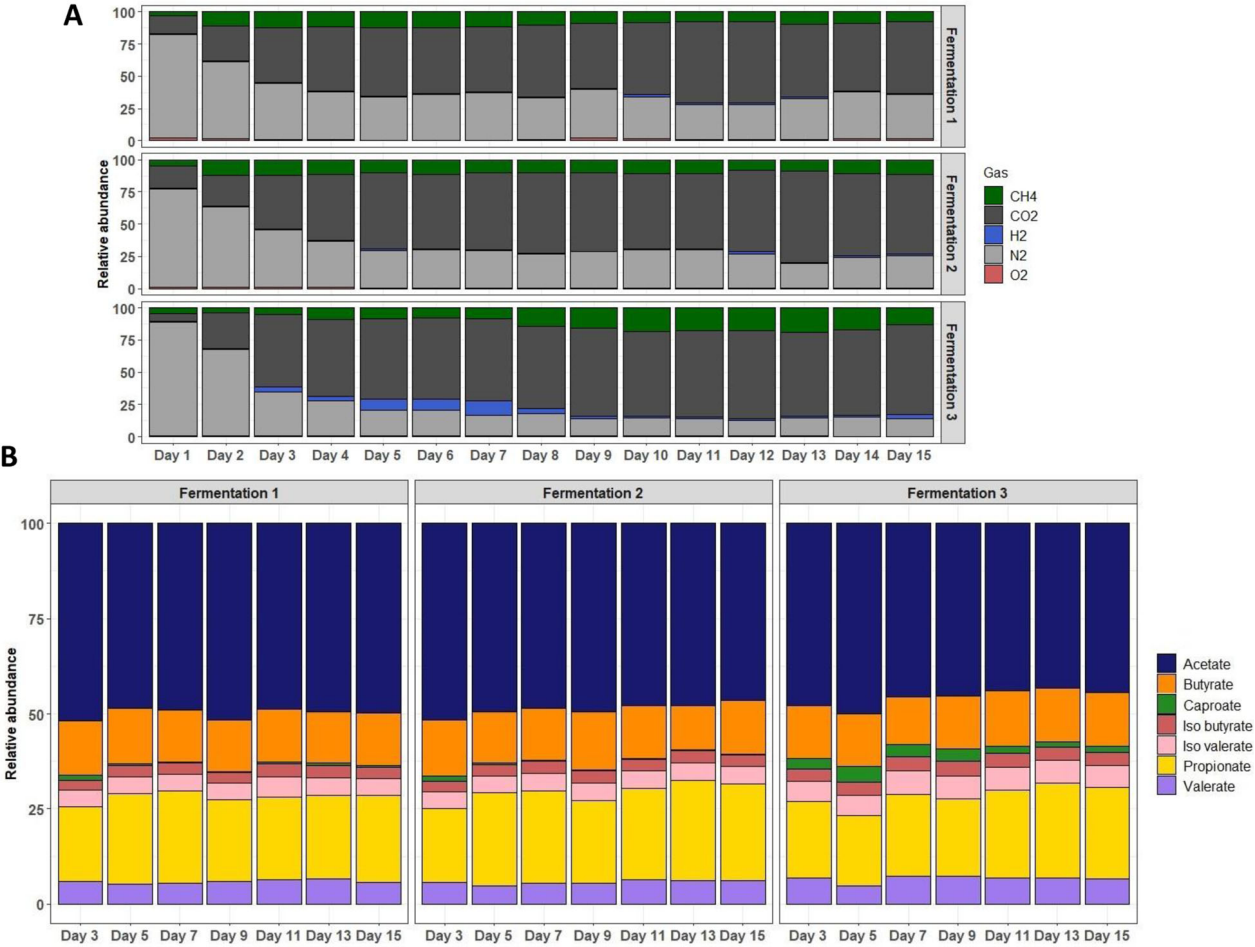
Set-up and validation of the MPigut-IVM: microbiota activity. **a** Relative abundance of gas produced by fermentation activity of the microbiota inhabiting the MPigut-IVM during control assays. **b** Short chain fatty acids (SCFA) relative abundance produced by fermentative activity of the microbiota inhabiting the MPigut-IVM during control assays in the bioreactor medium

Mean percentages of H_2_, O_2_, CO_2_, N_2_ and CH_4_ were respectively equal to 1.6 ± 2, 0.8 ± 0.5, 61.5 ± 5.7, 24.5 ± 8.4 and 11.7 ± 3.7 for the fermentations #1, 2 and 3 (Fig. 2a).

The general SCFA profile remained close to that measured in the fecal inoculum (Supplementary Figure 2A) and displayed mean relative percentages of 57.9 ± 1.2, 26.2 ± 1.2 and 16 ± 0.3 respectively for acetate, propionate and butyrate (Fig. 2b). The average total SCFA concentration from day 7 to day 15 was 259.35 ± 16.59 mmol/L for the fermentations #1, 2 and 3.

#### The MPigut-IVM harbors an abundant and taxonomically diversified microbiota

**Fig. 3:**
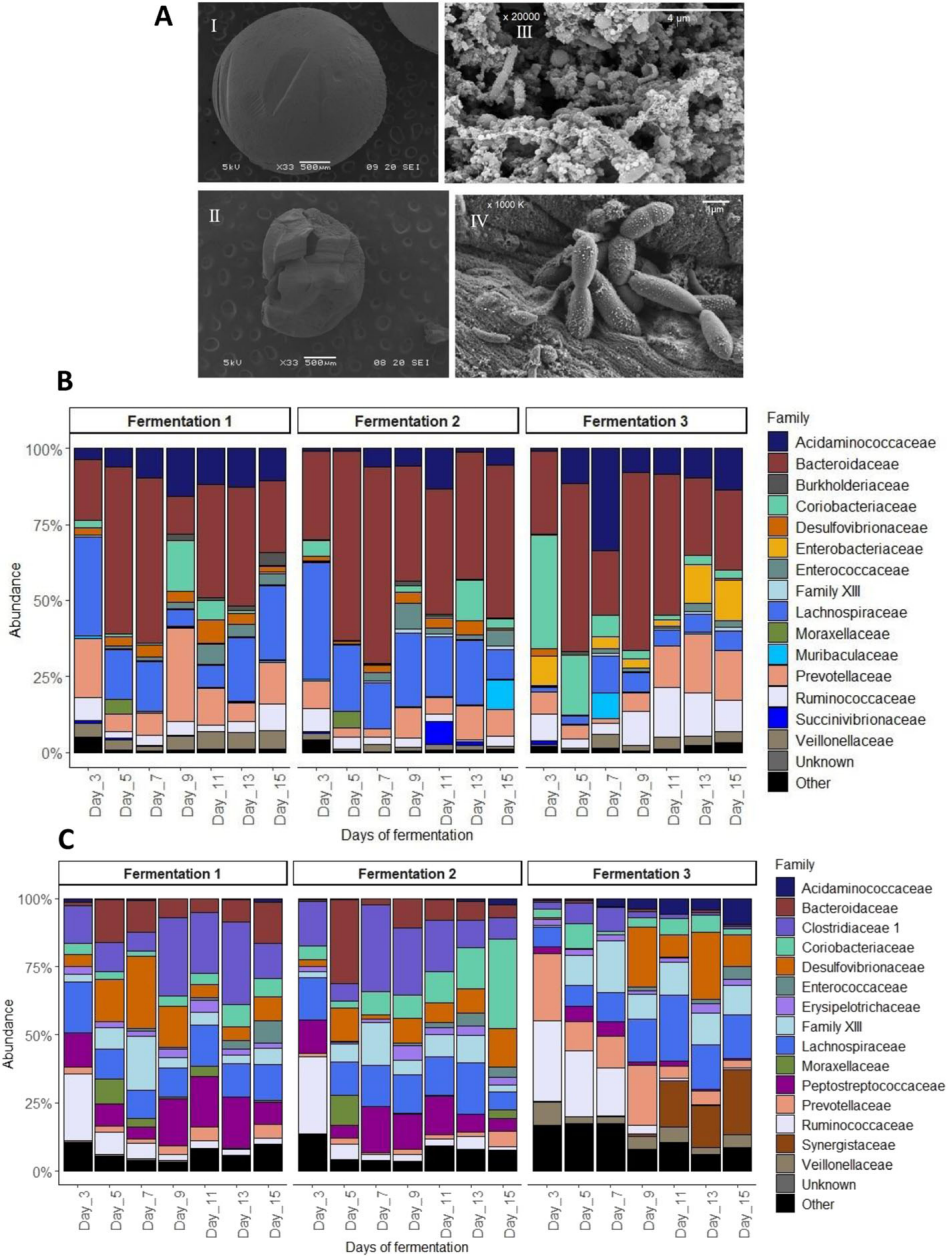
Set-up and validation of the MPigut-IVM: microbiota composition. **a** Evolution of the structure and colonization of a mucin bead before (I) and after (II) 48 h of incubation in the MPigut-IVM during the fermentation #1 and observation of the specific adherent microbiota at high magnitude (III & IV) by scanning electron microscopy. **b** Relative abundance of the 15 principal bacterial families in the bioreactor medium of the MPigut-IVM during control assays measured by 16S Illumina sequencing. **c** Relative abundance of the 15 principal bacterial families on the mucin beads of the MPigut-IVM during control assays measured by 16S Illumina sequencing

SEM observation of mucin beads incubated during 48 h, corresponding to the time mucin beads stayed in the mucin bead compartment of the MPigut-IVM, confirmed the presence of an abundant adherent microbial population at the surface of the beads. A strong degradation of the bead surface was observed compared with non-incubated mucin beads (Fig. 3a).

**Fig. 4:**
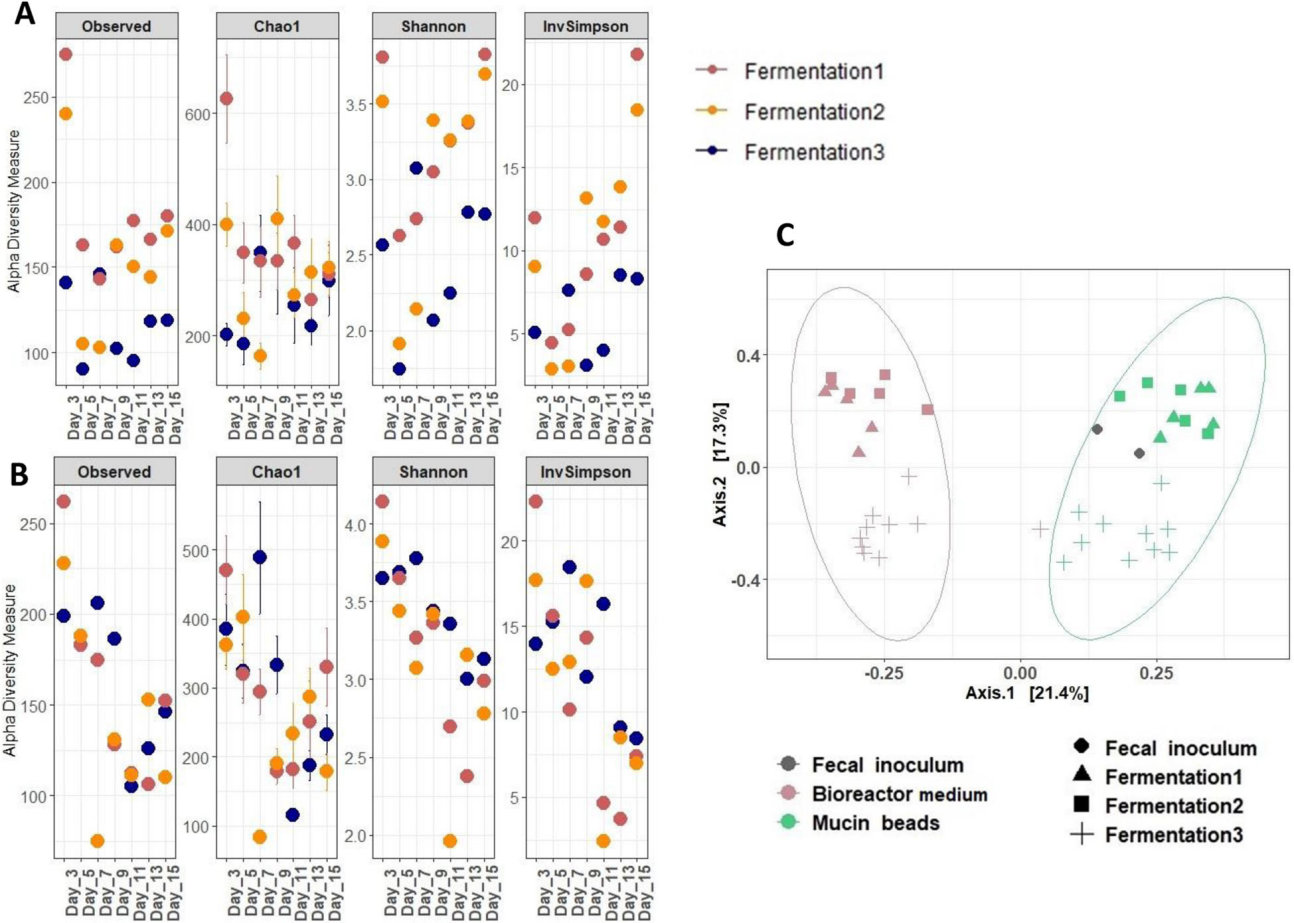
Set-up and validation of the MPigut-IVM: microbiota diversity. **a** Alpha diversity indices based on bacterial OTUs in the bioreactor medium of the MPigut-IVM during the fermentations #1, 2 and 3. **b** Alpha diversity indices based on bacterial OTUs on the mucin beads of the MPigut-IVM during the fermentations #1, 2 and 3. **c** Principal component analysis (PCoA) plot with Bray-Curtis dissimilarity on the bacterial communities in the bioreactor medium and on the mucin beads of the MPigut-IVM from the end of stabilization phase (day 7) to the end of fermentation (day 15) of the fermentations #1, 2 and 3

QPCR results showed that compared to fecal inoculum, Bacteroidetes level was increased in the bioreactor medium (Fig. 4a & Supplementary Figure 2B). After day 7, Firmicutes, Bacteroidetes*, Escherichia/Shigella* group, *Clostridium* cluster IV and *Clostridium* *cluster* XIVa populations were quantified at an average of 10.8 ± 0.4, 10.3 ± 0.4, 10.2 ± 0.4, 7.6 ± 1.3, 6 ± 0.7 and 10.2 ± 0.6 log_10_ copies of 16S rDNA per g of samples, respectively, in the bioreactor medium for the fermentations #1, 2 and 3. On mucin beads, after stabilization of populations, Firmicutes, Bacteroidetes*, Clostridium* cluster IV and *Clostridium* cluster XIVa groups mean abundances in mucin beads were of 9.2 ± 0.4, 8.2 ± 0.3, 8.7 ± 0.8 and 6.7 ± 1.2 log_10_ copies of 16S rDNA per g. *Clostridium* cluster XIVa population dramatically decreased across time. The level of total bacteria in the mucin beads was on average at 9.4 ± 0.3 log_10_ copies of 16S rDNA per g, lower than the 10.8 ± 0.4 log_10_ copies of 16S rDNA per g of total bacteria observed in the bioreactor medium (Fig. 4b & Supplementary Figure 3).

Concerning the 16S sequencing analyses, the most abundant bacterial phyla in the bioreactor medium of MPigut-IVM were Bacteroidetes (51.3% ± 11.2%), Firmicutes (37.6% ± 9.5%)*,* and Proteobacteria (6.5% ± 3.9%) (Supplementary Figure 4A). On the mucin beads, the Firmicutes mean relative abundance was 72.1% ± 12.7%, followed by Proteobacteria at 10.2% ± 6.7%, followed by the Bacteroidetes and Actinobacteria phyla at respectively 7.7% ± 4.9% and 6.8% ± 4.6%, from day 7 to day 15 (Supplementary Figure 4B). At a lower taxonomic level, the MPigut-IVM was principally composed of Bacteroidaceae*,* Lachnospiraceae, Prevotellaceae and Acidaminococcaceae families in the bioreactor medium whereas some families predominant in the fecal inocula were exclusively found in the mucin beads such as Clostridiaceae 1, Peptostreptococcaceae and *Clostridium* Family XIII (Fig. 3b & c). Interestingly, an archaeal community was successfully maintained in the MPigut-IVM and was exclusively composed of members of the Euryarchaeota phylum including the families Methanobacteriaceae and Methanomethylophilaceae (Supplementary Figure 5). The fecal inocula were also exclusively composed of these two archaeal families (Supplementary Figure 2).

During the first 5 d, the Shannon diversity index decreased from a mean value of 4 in the fecal inocula (Supplementary Figure 2F) to mean values from 2.9 to 3.4 from day 7 until day 15 in the bioreactor medium. The observed number of OTUs were maintained at around 600 from day 7 to 15 in the bioreactor medium. On mucin beads, the observed number of OTUs and the Shannon index ranged respectively between 300 and 600 and between 2.4 and 3.5 from day 7 to the end of the fermentation runs. Principal coordinate analysis (PCoA) on Bray Curtis distance showed that communities from mucin beads/fecal inocula and from bioreactor medium formed distinct clusters (Fig. 2h).

#### The stabilization phase is achieved after 7 days of fermentation

In *in vitro* continuous fermentation models, it is necessary to set up a stabilization period during which no treatments should be applied in order to make the transition from *in vivo* to *in vitro* conditions as well as the transition from a fecal to a colonic environment. Regarding the quantification of bacterial populations, the measurement of SCFA, gas composition, and the Illumina sequencing data of the fermentations #1, 2 and 3 stated above, a stabilization period of 7 d was the best compromise for the MPigut-IVM. Additionally, percentages of variation between the day 0 to 3, 3 to 5 and 5 to 7 were calculated using the data of SCFA, gas composition and the MPigut-IVM top 10 families abundance by sequencing. These percentages of variation were below 5% for the day 5 to 7 which was considered as satisfying to set up the end of the stabilization phase at day 7 (Supplementary Figure 6).

#### The microbiota of the MPigut-IVM is close to the in vivo microbiota of piglet proximal colon

**Fig. 5:**
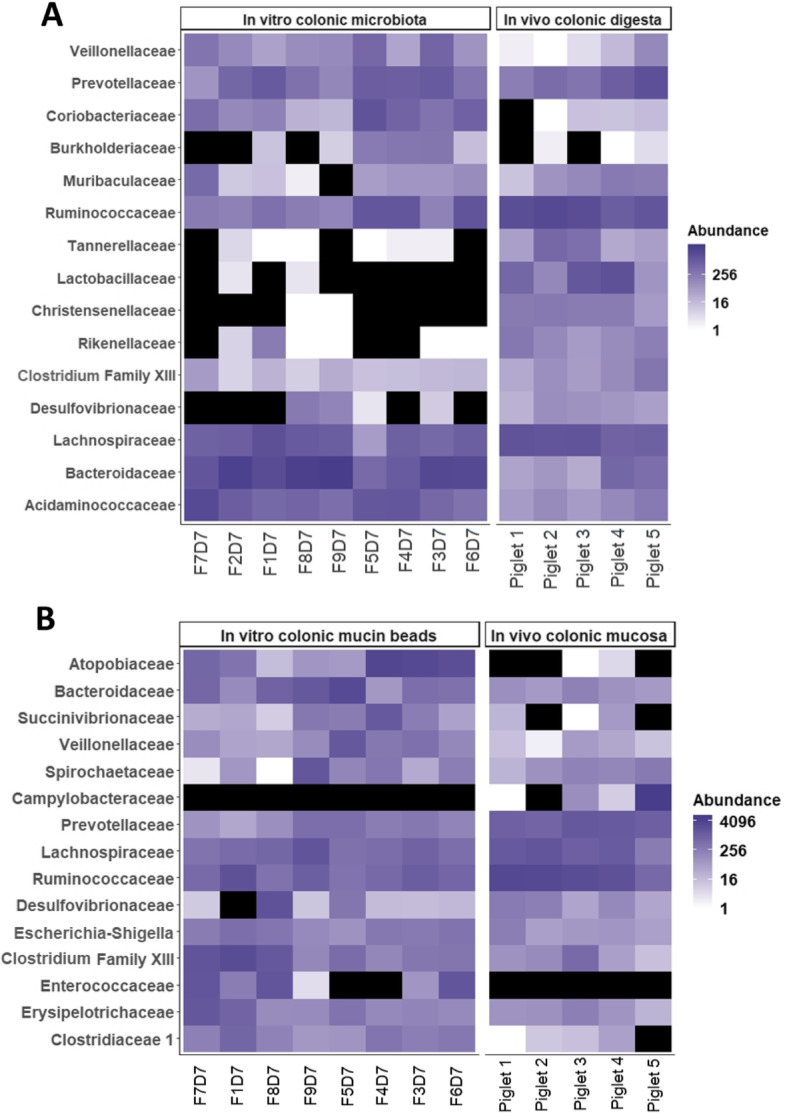
Set-up and validation of the MPigut-IVM: comparison with *in vivo* data. I. Comparison of the top 15 bacterial families between *in vivo* proximal colon luminal samples and *in vitro* fermentation samples from the bioreactor medium at day 7 corresponding to the end of the stabilization phase. The black colour corresponds to an abundance of 0. On the X axis: “F” = “Fermentation” and “D” = “Day”. J. Comparison of the top 15 families between *in vivo* proximal colon mucosal samples and *in vitro* mucin beads at day 7 corresponding to the end of the stabilization phase. The black colour corresponds to an abundance of 0. On the X axis: “F” = “Fermentation” and “D” = “Day”

The abundance of the 15 top bacterial families from all the bioreactor medium and mucin beads samples at day 7 were compared to *in vivo* colonic luminal and mucosal samples from 5 healthy 28 days old piglets. In the bioreactor medium, the most abundant retrieved families, in particular Veillonellaceae, Prevotellaceae, Muribaculaceae, Ruminococcaceae, Lachnospiraceae, Bacteroidiaceae and Acidaminococaceae were those also found in the *in vivo* colonic samples. Tannerellaceae, Lactobacillaceae and Christensenellaceae seemed to be overrepresented in the MPigut-IVM compared to *in vivo* (Fig. 5a). The *in vitro* microbiota communities of mucin beads showed a high similarity with *in vivo* samples from the proximal colon mucosa of piglets as visualized through Heatmaps (Fig. 5b). Nevertheless, the Atopobiaceae and Enterococcaceae displayed a higher abundance, and Campylobacteraceae lower relative abundance, in *in vitro* mucin beads compared with *in vivo* mucobiome. Analysis of the *in vivo* microbiota also highlighted variability between individuals which is kept in the MPigut-IVM (Fig. 5a and b). To steer the comparison between *in vivo* and *in vitro* bacterial communities a PCoA plot with Bray Curtis dissimilarities is presented in Supplementary Figure 7.

### A feed deprivation stress triggers microbial dysbiosis in the MPigut-IVM

In order to better understand the etiology of microbial dysbiosis in post-weaning piglets, the effects of a feed deprivation stress were evaluated using the newly developed MPigut-IVM.

#### Effects of 12 h, 24 h and 48 h feed deprivation stresses on the microbial composition

**Fig. 6:**
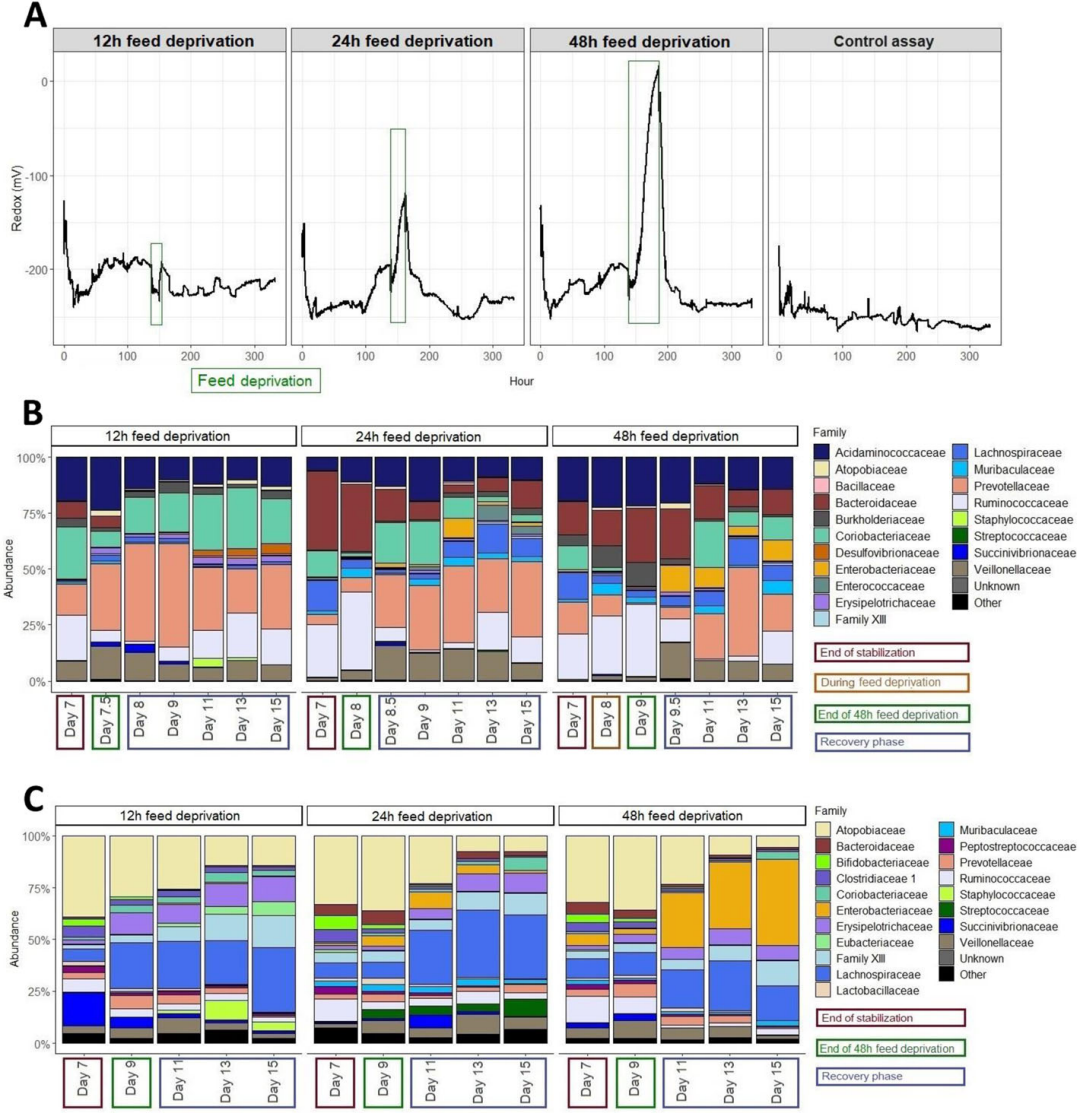
Effects of a feed deprivation stress of 12, 24, and 48 h on the MPigut-IVM microbiota composition. On this figure, the “12h feed deprivation stress”, “24h feed deprivation stress” and “48h feed deprivation stress” correspond to the fermentations #4, 5 and 6, respectively. **a** Evolution of the redox potential. An example of redox measurement for a control assay is provided one the figure. These data are from the fermentation 2. **b** Effect of a feed deprivation period of 12, 24 or 48 h on the relative abundance of the main bacterial families in the bioreactor medium of the MPigut-IVM as measured by 16S Illumina sequencing. **c** Effect of a feed deprivation period of 12, 24 or 48 h on the relative abundance of the main bacterial families on the mucin beads of the MPigut-IVM as measured by 16S Illumina sequencing

The fermentations #4, 5 and 6 were performed using the same fecal inoculum and were subjected to feed deprivation periods of 12, 24 or 48 h, respectively. Due to the absence of replicates, no statistics were performed on this section. Applications of gradual feed deprivation stress induced progressive modifications of the redox potential and bacterial composition. For instance, a gradual increase of Enterobacteriaceae was measured by qPCR and 16S sequencing both on the mucin beads and the bioreactor (Fig. 6 and Supplementary Figure 8).

#### A 48 h feed deprivation stress impacts the in vitro microbiota composition and activity

The fermentations #6, 7, 8 and 9 were carried out with different fecal inocula (*n* = 4) and were subjected to a 48 h feed deprivation from day 7 to 9 of the fermentation runs.

##### QPCR quantification of bacterial and archaeal groups

**Fig. 7:**
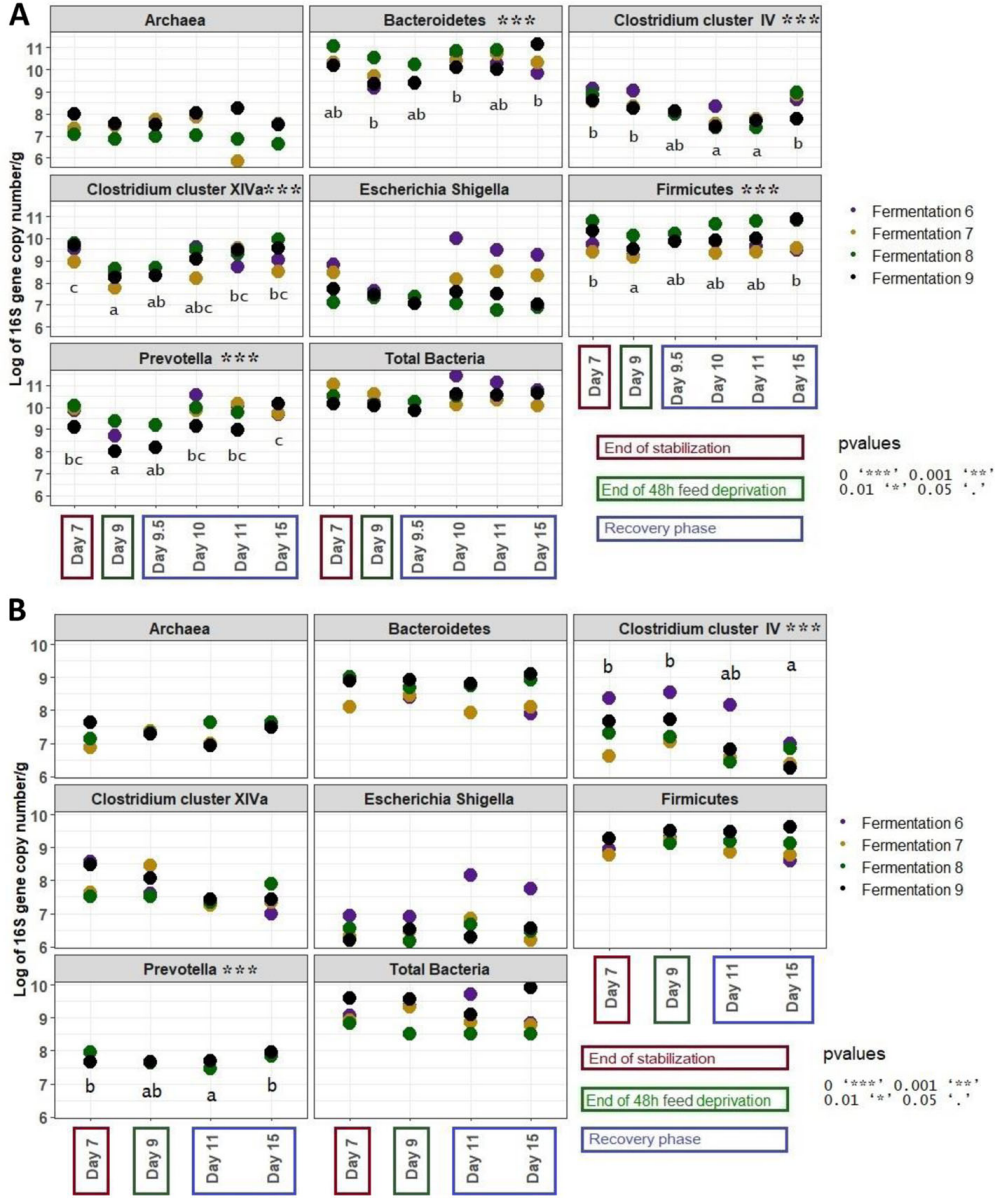
Quantification of bacterial and methanogen archaeal in the bioreactor medium (**a**) and on the mucin beads (**b**) of the MPigut-IVM during the fermentations #6, 7, 8 and 9 which were subjected to a feed deprivation stress of 48 h (*n* = 4 for each time point)

In the bioreactor medium, compared to day 7, *Prevotella, Clostridium* cluster IV and Firmicutes were significantly reduced from day 9 to 11, and *Clostridium* cluster IV and Bacteroidetes were significantly reduced by from day 9 to day 15 (Fig. 7a). The abundance of the *Escherichia-Shigella* group was quite variable between the four fermentation runs but a clear augmentation of the abundance of this group from 7.6 and 7.4 at day 9 to 9.3 and 8.3 at day 15 respectively in the fermentations #6 and 7.

On mucin beads, compared to day 7, *Prevotella* group was significantly reduced at day 9 and *Clostridium* cluster IV displayed significantly lower values at day 11 and even at day 15. *Escherichia-shigella* group was increased on the mucin beads at day 11, though not significantly (Fig. 7b).

##### Effects on the *in vitro* microbiota using Illumina MiSeq sequencing technology

**Fig. 8:**
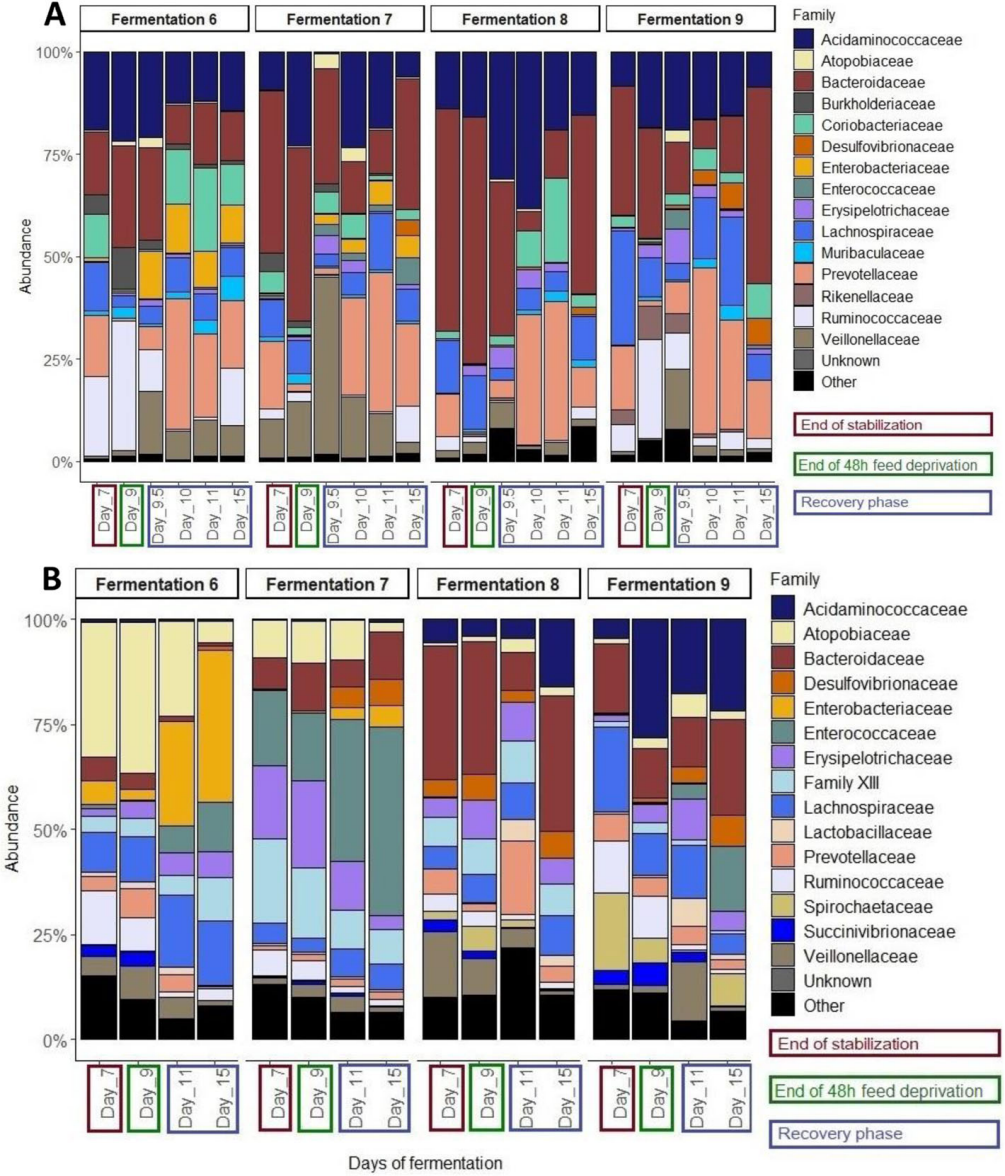
Relative abundance of the 15 main bacterial families in the bioreactor medium (**a**) and on the mucin beads (**b**) of the MPigut-IVM during the fermentations #6, 7, 8 and 9 which were subjected to a feed deprivation stress of 48 h, as measured by 16S Illumina sequencing

The number of sequences generated by the Illumina MiSeq run are presented in Supplementary Table 3 while the results of post hoc statistical tests performed on Illumina sequencing data are presented in Supplementary Table 4. Firmicutes relative abundance increased from 40.4 ± 9% to 50.4 ± 10.4% and Bacteroidetes relative abundance decreased significantly from 51.2 ± 14.2% to 43.7 ± 14.5% from day 7 to day 9.5. On the mucin beads, no significant differences were observed at the phylum level (Supplementary Figure 9B). Compared to day 7, relative abundance of Ruminococcaceae family was significantly reduced after the feed deprivation period (day 10 and 11), Prevotellaceae family was significantly increased after the feed deprivation period (day 10), Bacteroidiaceae was significantly decreased at days 10 and 11, Veillonellaceae were significantly increased after the feed deprivation period at day 9.5, and Coriobacteriaceae and Atopobiaceae families were significantly less abundant respectively at day 9 and 9.5. At day 15, the previously cited families recovered values similar to day 7 (Fig. 8a). The Lachnospiraceae family was significantly decreased at day 9.5 compared to day 7 but recovered normal values at day 10 to 15 (Fig. 8a).

On the mucin beads, the relative abundance of Peptostreptococcaceae, Clostridiaceae 1, Ruminococcaceae and Veillonellaceae families were lower from day 11 and day 15 compared to day 7 and day 9 (*P-*value < 0.05) (Fig. 8b).

The Enterobacteriaceae family relative abundance showed a strong increase both on the mucin beads and in the bioreactor medium during the whole recovery period (day 9.5 to day 15) of the fermentations #6 and 7 (Fig. 8a and b). Interestingly, this family was also more abundant in the fecal inocula of the fermentations #6 and 7 compared to the fecal inocula of the fermentations #8 and 9 (Supplementary Figure 2D).

**Fig. 9:**
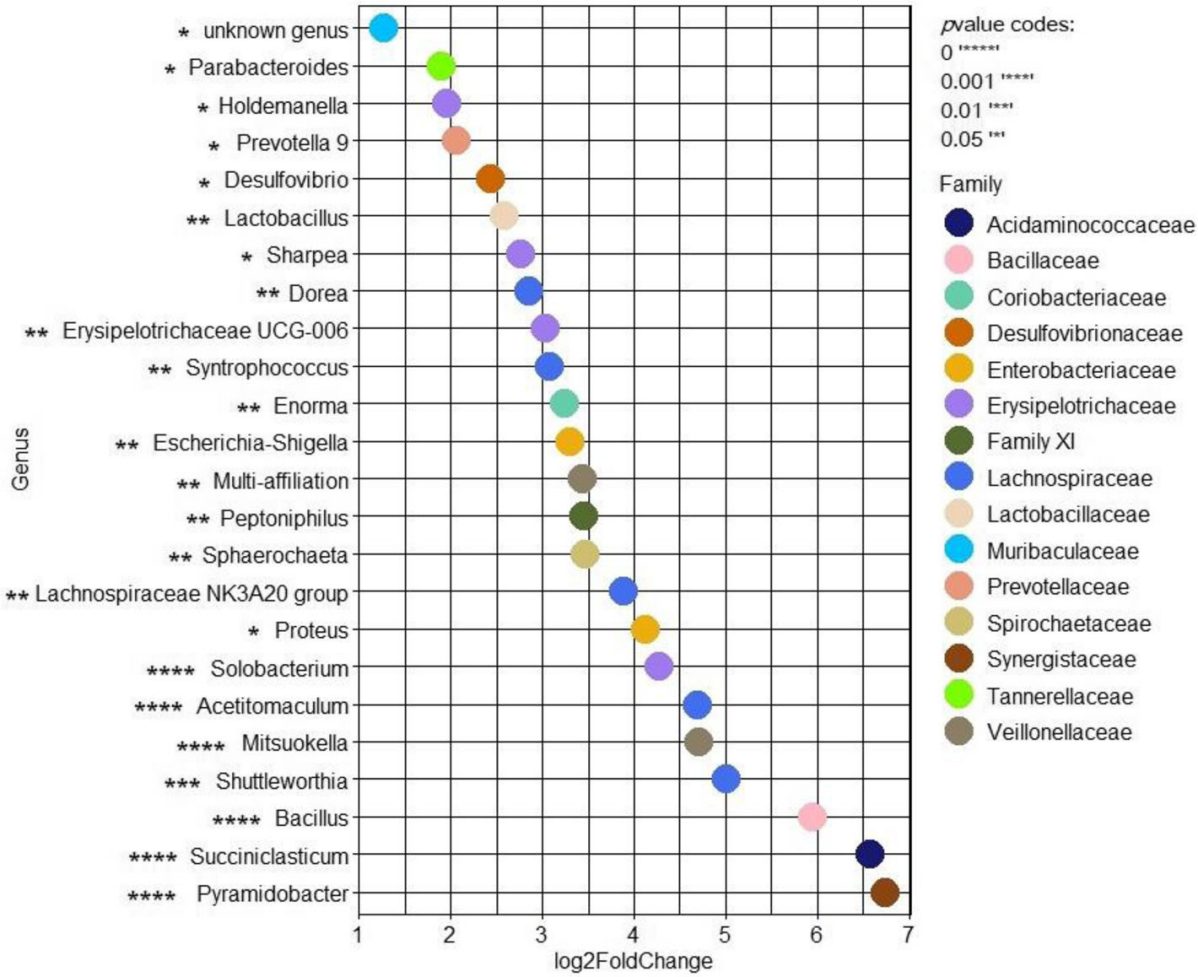
Differentially abundant genera between the end of the stabilization (day 7) and the recovery phase (day 9.5, 10, 11 and 15) in the bioreactor medium, mucin beads and bead medium of the fermentations #6, 7, 8 and 9. Only significative Log_2_FoldChanges are represented on the figure. *P-*value codes are indicated on the figure

Wald test analysis using DESEQ2 package between day 7 and all the time points of the recovery period showed that *Escherichia-Shigella* group was significantly more abundant after the feed deprivation stress along with *Pyramidobacter, Succiniclasticum, Bacillus, Mitsuokella* genera and several members of the Lachnospiraceae family, notably (Fig. 9).

Alpha diversity indices calculated for both the bioreactor medium and the mucin beads displayed variable values depending on the fermentation numbers but no statistical difference was observed (Supplementary Figure 12).

#### Effects of feed deprivation stress on microbial activity

##### Measurement of redox potential, gases and SCFAs

A 48 h feed deprivation stress induced a strong augmentation of the redox potential in the 4 fermentation runs to maximal values of + 17, − 21, − 94 and − 78 mV. In all bioreactors, the redox potential recovered a very low value (− 250 mV) within ~ 20 h after restarting of the feeding pump (Supplementary Figure 12). The feed deprivation period of 48 h in the MPigut-IVM led to modifications of gas composition with an slight augmentation of N_2_ and O_2_ percentages and a decrease of CO_2_ percentage, which was not attributed to any leak of the system (Supplementary Figure 13).

**Fig. 10:**
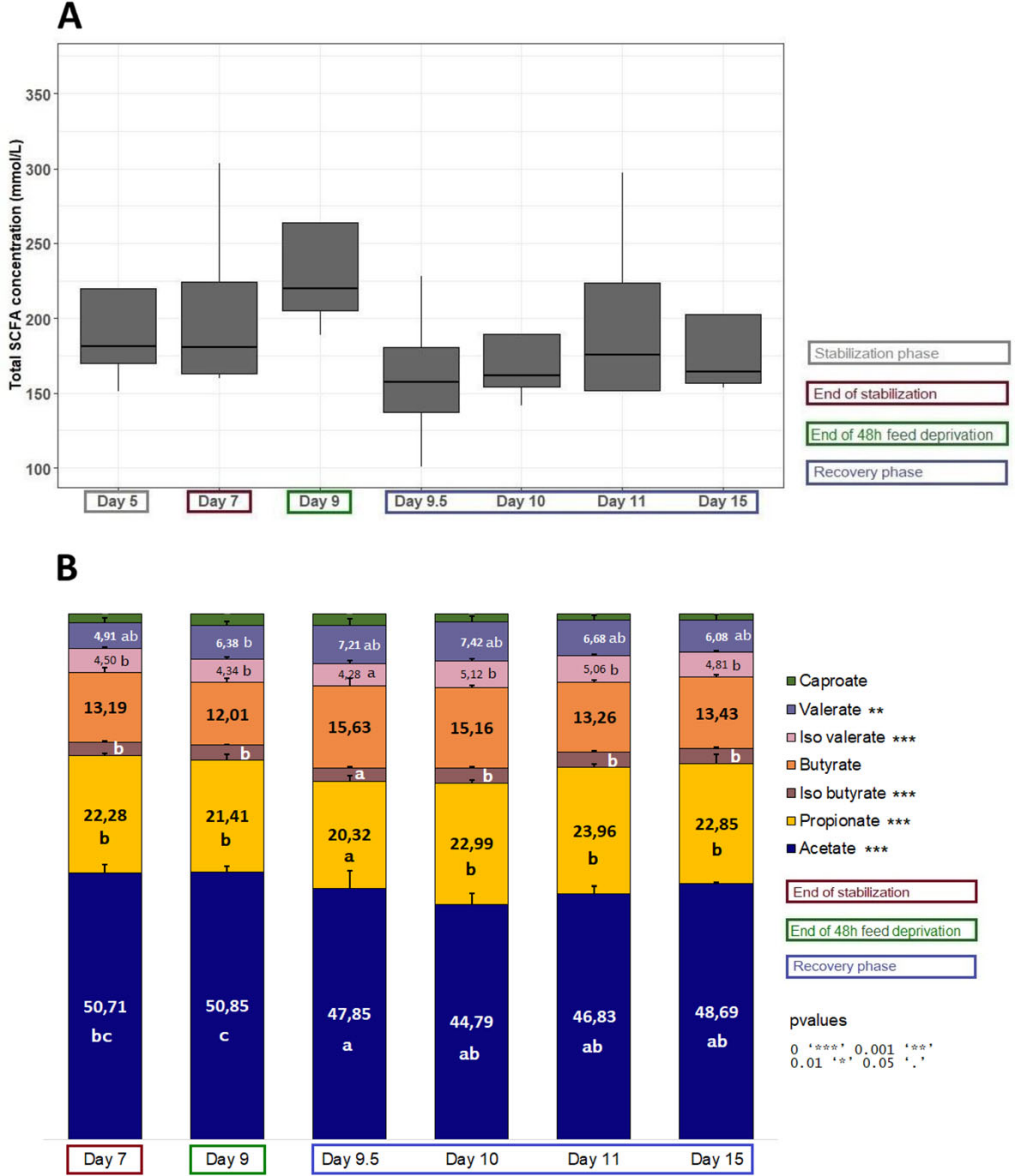
Evolution of the mean total concentration (**a**) and of the relation abundance (**b**) of SCFAs during the fermentation #6, 7, 8 and 9 which were subjected to a feed deprivation stress of 48 h (*n* = 4 for each time point)

Compared to day 7, total SCFA concentration decreased, though not significantly, at day 9 (Fig. 10a). ANOVA analyses revealed that, compared to day 7, acetate percentage was significantly reduced from day 9.5 to day 15, valerate was significantly increased at day 9 while propionate, isobutyrate and isovalerate were significantly reduced at day 9.5 (Fig. 10b).

##### Metabolome analysis using NMR

**Fig. 11:**
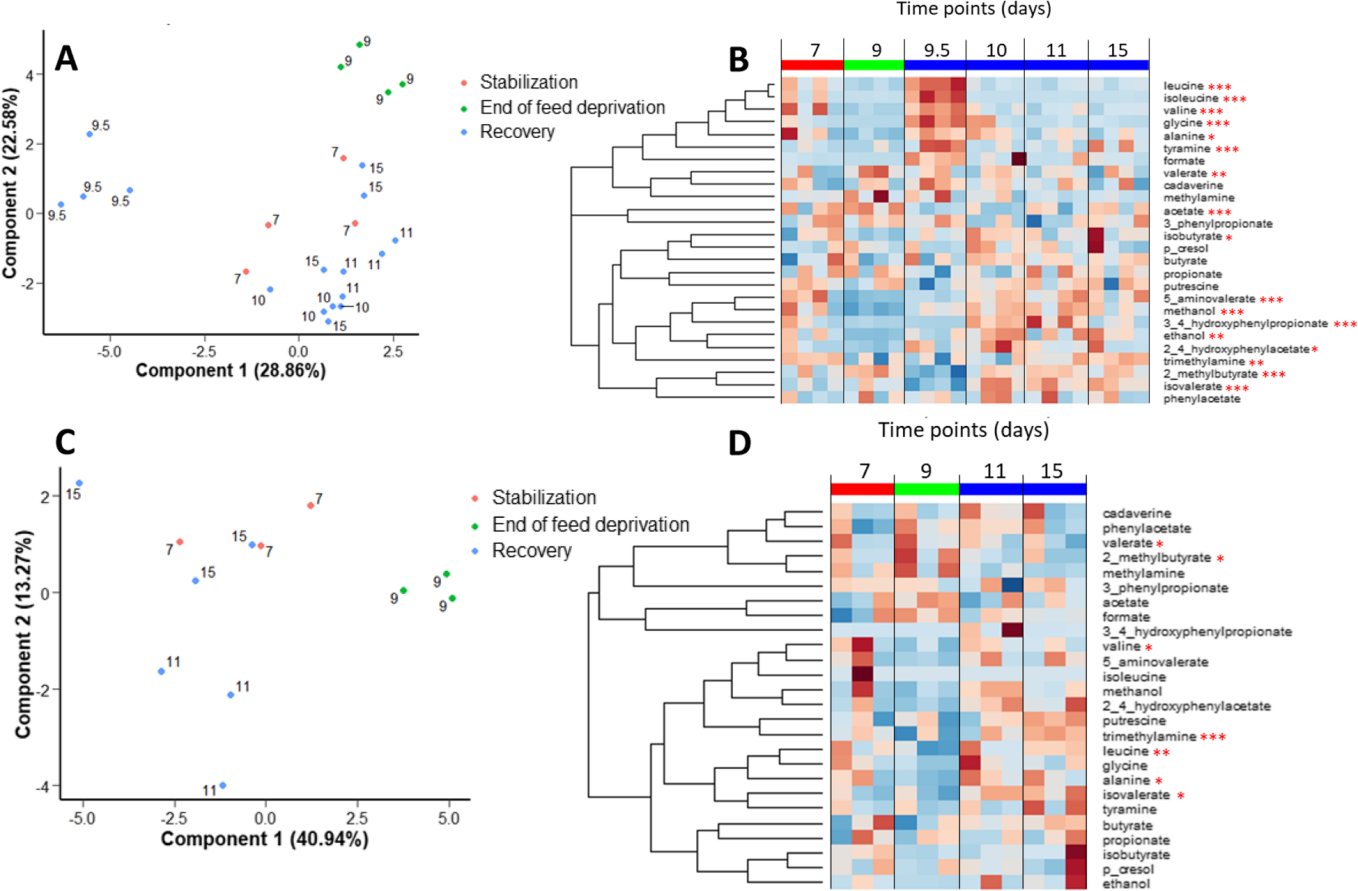
Analysis of the *in vitro* metabolome by nuclear magnetic resonance (NMR). Metabolomics analysis were performed by using NMR in the bioreactor medium (**a** and **b**) and bead medium (**c** and **d**) compartments. Individual plots of partial least square-discriminant analysis using metabolites as variables and time points as predictors (**a** and **c**). Heatmap representing the relative concentrations of all identified metabolites (rows) in individual samples (columns). The color represents the Z-scores (row-scaled relative concentrations) from low (blue) to high (red) values. Metabolites (rows) were clustered by the average method. The mean relative abundances were analyzed by a mixed model and Anova.*: *P* < 0.05, **: *P* < 0.01, ***: *P* < 0.001 (adjusted *P*-values by the false discovery rate method). The bead medium of the fermentation number 6 was not analyzed by NMR which explains the three replicates for the figure **c**

Overall, 26 metabolites could be identified (Supplementary Table 5). PLS-DA revealed a dynamic remodeling of the metabolome in the bioreactor medium according to the stabilization, feed deprivation stress and recovery periods (Fig. 11a and b). In the bioreactor medium, at day 9 compared to day 7, the concentrations of methanol, trimethylamine, 5-aminovalerate and of the amino acids alanine, valine and leucine were decreased while valerate and 2-methylbutyrate concentrations increased. At day 9.5, a strong increase in glycine, alanine, isoleucine, leucine, valine and tyramine concentrations and a decrease of isovalerate, acetate and 2-methylbutyrate was observed. At later time points (days 10–15), the concentration of amino acids decreased (glycine, isoleucine, leucine and valine) while the concentration of the bacterial metabolites 3–4-hydroxyphenylpropionate and isovalerate increased (Fig. 11a and b, Supplemental Table 6).

In the bead medium, PLS-DA also revealed that the 48 h feed deprivation decreased significantly the concentration of trimethylamine, valine and leucine. From day 9.5 to 15, the concentration of trimethylamine, isovalerate, 2-methylbutyrate, alanine and leucine gradually increased while methylamine and valerate decreased (Fig. 11c and d, Supplementary Table 6).

### Metabolome-microbiome Spearman’s correlation

**Fig. 12:**
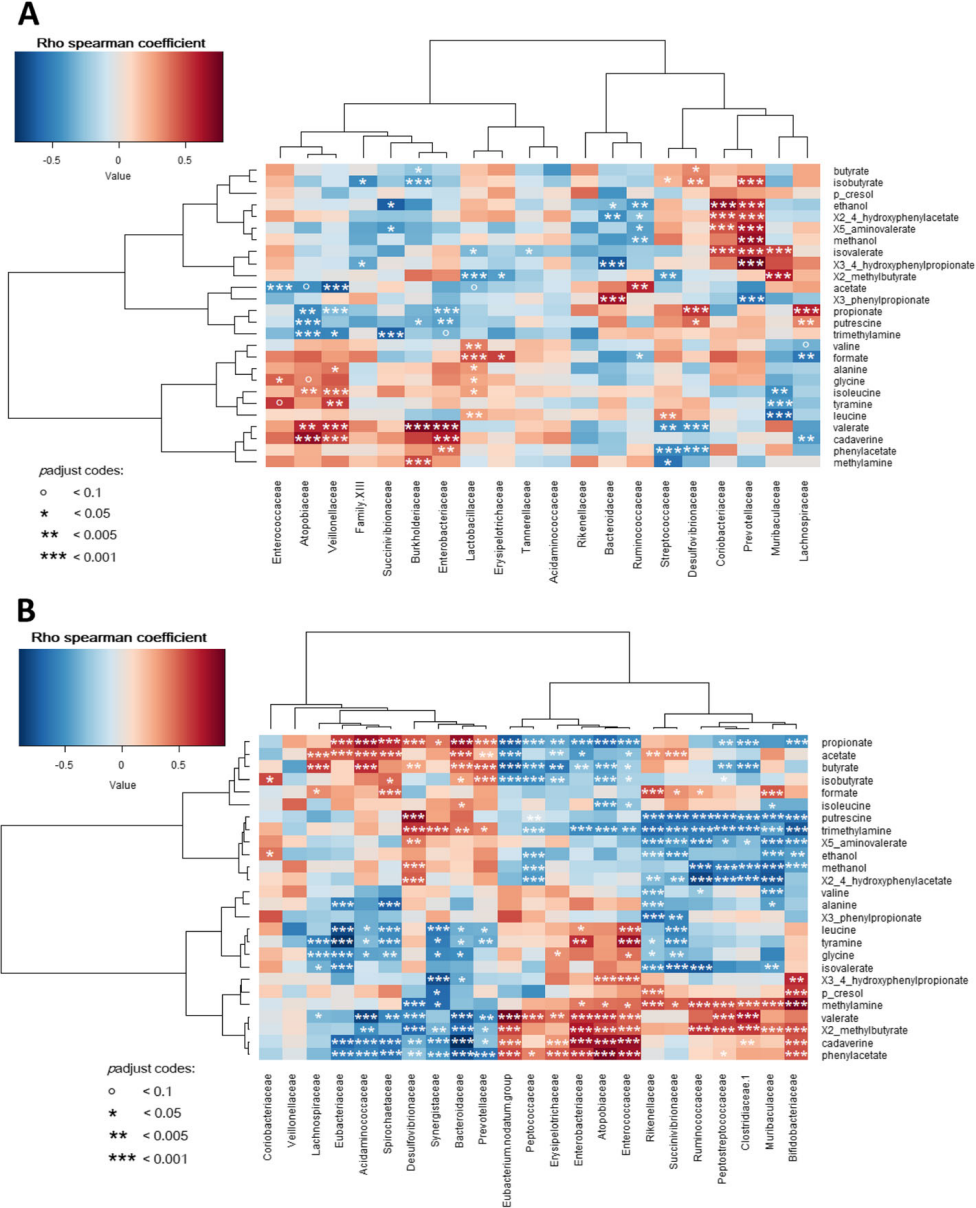
Spearman's correlation between the relative abundance of the main bacterial families and the metabolites in the bioreactor medium (**a**) and on the mucin beads (**b**) of the MPigut-IVM during the fermentation 6, 7, 8 and 9 at day 7, 9, 9.5, 10, 11 and 15. Cells are colored based upon the Spearman correlation coefficient. Asterisks indicate significant *P* values (< 0.05)

In the bioreactor medium (Fig. 12a), the strongest positive Spearman correlation [*ρ* (correlation coefficient) > 0.7, *P* < 0.005] were between valerate, cadaverine and Enterobacteriaceae, between Prevotellaceae and hydroxyphenylpropionate, methanol, and aminovalerate, and between Bacteroidiaceae and phenylpropionate. Leucine was strongly negatively correlated with the Muribaculaceae family (*ρ* < − 0.8, *P* < 0.005).

In the bead medium (Fig. 12b), valerate was positively correlated (*ρ* > 0.8, *P* < 0.005) with *Eubacterium nodatum* group, Clostridiaceae 1, Peptostreptococcaceae and Enterobacteriaceae and negatively correlated (*ρ* < − 0.8, *P* < 0.005) with Bacteroidaceae, Acidaminococcaceae and Prevotellaceae*.* Butyrate and methyl butyrate were positively associated with Acidaminococcaceae and Clostridiaceae 1. Propionate and phenylacetate were respectively positively correlated (*ρ* > 0.8, *P* < 0.005) with Bacteroidaceae/Acidaminococcaceae and Atopobiaceae*/*Enterococcaceae families. Biogenic amines, methylamine and trimethylamine, were positively correlated (*ρ* > 0.8, *P* < 0.005) with Bifidobacteriaceae. At least, cadaverine could be positively correlated (*ρ* > 0.8, *P* < 0.005) with Atopobiaceae*,* Enterobacteriaceae and Enterococcaceae and negatively correlated (*ρ* < − 0.8, *P* < 0.005) with notably Bacteroidaceae*.* Putrescine was positively correlated with Desulfovibrionaceae (*ρ* > 0.8, *P* < 0.005) and negatively correlated (*ρ* < − 0.8, *P* < 0.05) with Muribaculaceae*,* Peptostreptococcaceae and Ruminococcaceae*.*

## Discussion

We developed a novel *in vitro* technology to accurately mimic the piglet gut. As opposed to existing models [21, 24, 47, 48], the MPigut-IVM possesses a unique multiparametric set up by incorporating a mucus environment, an independently established anaerobic redox condition, a fine-tuned and representative piglet feed and a physiologically representative residence time [27]. Our newly developed MPigut-IVM shows good reproducibility between replicates while maintaining some inter-inocula specificities and displays consistent shifts from a fecal to a colon microbiota such as the increase of the Bacteroidetes phylum [26]. The augmentation of Bacteroidetes and Proteobacteria relative abundance is commonly observed in *in vitro* models due to initial quite high oxygen concentration and reactor startup [20, 21, 48–50]. We could notice that very few differences were observed in the microbiota composition between technical replicates of control assays (fermentations #1 and 2) and sampling time, compared with the biological replicate (fermentation #3). This shows the reproducibility of *in vitro* results when using a similar fecal sample for inoculation but also that MPigut-IVM was able to reproduce inter individual differences that can be generally observed in animal experiments. This is a great advantage to take into account some variability in order to mimic even more closely *in vivo* conditions and thereby get reliable results when testing the influence of biotic or abiotic factors on colonic microbial environment. Part of the variability observed between biological replicates can be explained by the use of fresh fecal material. Indeed, freezing of the fecal samples allows the preparation of a standardized inoculum to ensure better reproducibility and good comparison between studies . However, Metzler-Zebeli et al. demonstrated that freezing pig feces could influence the relative abundance of *Lactobacillus, Enterococcus, Streptococcus, Clostridium, Bacteroides, Prevotella* and Enterobacteriaceae analysed by 16S sequencing [25]. Therefore, if freezing influences results from DNA-based downstream analyses, we can easily imagine a strong impact on bacteria functions and cultivability in *in vitro* models. A study of Pastorelli et al. confirmed that fresh or frozen pig fecal inocula induced differences on *in vitro* fermentation capacities for degradation of various feed ingredients [51]. The use of pooled fecal samples was due to the impossibility, regarding the size of the animals, to obtain enough fecal material from one single donor to start several bioreactors run in parallel. In the past, starting *in vitro* fermentation from a pool of feces was questioned due to the hypothesis that the obtained microbiota would be reflecting only the most dominant species as a result of some competition for ecological niches [52]. Yet, several studies comparing the use of a pool of feces with inocula from single donors in *in vitro* models provided contradictory results by showing that pooled microbiota was able to reflect similar metabolic profile and bacteria composition compared to what was observed from a single donor [53, 54].

The principal characteristics of the MPigut-IVM, compared to other *in vitro* models, is the tailor-made mucin bead compartment aiming to reproduce the microbiota associated with the mucus layer by allowing adhesion to mucins and their degradation. In *in vitro* models, it is impossible to represent the extensive and extremely thin area from the *in vivo* mucus layer unless working with a very small volume of beads which would be very quickly degraded and thus unavailable for analysis. The most important function was to have a mucosal adhesion platform to study the specificity of mucosal microbiome and its dynamics. The bead diameter allowed an efficient microbial colonization and a reproducible degradation of the mucin beads incubated for 48 h in the MPigut-IVM and thus, the beads were replaced every 48 h. Additionally, observations by scanning electron microscopy confirmed that 48 h was the appropriate time to observe a biofilm of strongly adherent microbiota on the mucin beads. Even though at each bead exchange, the fermentation medium located in the bead compartment and containing mainly degraded beads was conserved to inoculate the newly introduced beads, bead exchange every 48 h could however be responsible of the differences observed in alpha diversity by the loss of sub-dominant populations. In agreement with *in vivo* data [26, 55], the microbiota composition of the MPigut-IVM mucin beads was clearly distinct from that of the bioreactor medium and displayed a higher relative abundance of Proteobacteria and reduced Bacteroidetes compared to luminal microbiota, here simulated by the bioreactor medium [26, 55]. Erysipelotrichaceae, Peptostreptococcaceae, Clostridiaceae 1 and *Clostridium* Family XIII were more abundant in the mucin beads compared to the bioreactor medium, hypothetically due to their mucin-binding or mucin-degrading capacities. At least, the comparison between the fifteen most abundant families in mucin beads and our mucosal proximal colon samples clearly showed very similar profiles indicating a well-established and specific mucin-associated bacterial community in the MPigut-IVM.

Additionally, the MPigut-IVM was able to maintain an archaeal microbiota, composed of Methanobacteriaceae and Methanomethylophilaceae consistently with *in vivo* data [26], which was hitherto not observed in any other *in vitro* model [20, 21, 24]

Regarding microbial activity, methane was detected during the fermentation process indicating the presence of an active methanogenic archaea community, in agreement with *in vivo* observations in piglet [56, 57]. In comparison with the proportion of acetate, propionate and butyrate in the fecal inoculum, the *in vitro* relative abundance of acetate and butyrate were respectively slightly decreased or increased after the stabilization period of 7 d which was in concordance with our previous *in vivo* proximal colon data [26] and literature [58]. Besides SCFA, NMR metabolomics revealed that MPigut-IVM microbiota produced a wide diversity of metabolites such as biogenic amines (e.g. cadaverine, 5-aminovalerate, and putrescine) and aromatic metabolites (e.g. 3-phenylpropionate, phenylacetate, *p*-cresol). These results indicate that the functional capacity of the piglet gut microbiota is maintained *in vitro*.

Important MPigut-IVM characteristics were responsible of the *in vitro* microbiota shaping and could probably be even more improved such as simulation of ileal chyme via the nutritive medium or digestive secretions. However, data are very scarce in the literature or poorly described. Indeed, physico-chemical parameters of the gut are different depending on several criteria such as species or environment factors and should always be taken into account in order to make reliable comparison with *in vivo* situation*.* Information about transit time of the young pig is available in the literature [27]. However, this study didn’t discriminate proximal and distal colonic transit time. The set-up of the MPigut-IVM retention time was therefore based on the global colonic retention time measured on piglets from 35 d of age but could be better adjusted if new data are available. Regarding pH setup, we measured the pH of all the GIT compartments of weaning piglets, and the colonic pH values were all between 6 and 6.5 [26]. For the setup of the MPigut-IVM we chose to fix the pH to 6, the lower range of our *in vivo* data, in order to limit the expansion of the Bacteroidetes and Enterobacteriaceae which is most of the time observed in in vitro models during the stabilization period [21, 47]. The choice of nutritive medium components has of course also a great impact in modelling *in vitro* microbiota. For example, a nutritive medium which contains too many carbohydrates enhances the augmentation of Bacteroidetes which is often observed in *in vitro* models [59]. In the MPigut-IVM, a nutritive medium mimicking the ileal chyme of piglets was set up based on the protocols used by MacFarlane et al. [28] and Tanner et al. which calculated, using digestibility indices, the percentage of starch or proteins which should be not digested and absorbed by the host and reach intact the colon. The accuracy of some digestibility indices could be even more adapted to piglets of this age when such data will be available in the literature. The second point which was of importance was the widely spread practice of creep feeding which was taken into account by formulating a pre-weaning diet medium already containing vegetable proteins or fibre besides a high proportion of dairy product. Therefore, despite possible improvements, which will be implemented depending on the availability of data, the MPigut-IVM is able closely simulate piglet microbiota including individual as well as luminal versus mucosal differences. Thus the MPigut-IVM can be considered as an innovative and a powerful tool for comparative studies to investigate the impact of weaning stressors on piglet microbiota.

Once characterized, the MPigut-IVM was applied to evaluate the impact of weaning feed deprivation period, the most critical factor involved in the intestinal barrier dysfunction and potentially affecting the microbiota composition and function [15]. In the MPigut-IVM subjected to a feed deprivation stress, Bacteroidiaceae family was found significantly lower, while the Prevotellaceae increased in the bioreactor medium after the feed deprivation period compared to the end of the stabilization phase. Due to the selective distribution of the microbiota in the *in vitro* “luminal” and “mucus”-associated phases, a reduction of Clostridiaceae was observed on the mucin beads. The Clostridiaceae family contains numbers of mucin-adherent butyrate and acetate producers and is associated with a healthy microbiome [60]. Up to date, some researches have investigated the impact of weaning feed deprivation period on the intestinal microbiota and function of weaning piglets. Recent investigations have reported that deoxynivalenol (DON), the most common cereal crop mycotoxin, could induce anorexia and disruptions of the cecal and colon microbiota composition and SCFA concentration of 27 d old piglets [61]. In this study, the DON-induced anorexia was characterized by a significant decrease in genera belonging to Clostridiaceae and Bacteroidiaceae and a significant increase in members of Prevotellaceae [61]. *Pyramidobacter, Succiniclasticum, Bacillus* or *Shuttleworthia* genera, previously associated with production of acetate, iso-valerate, lactate or propionate [62–64], were significantly increased from more than 4.5 log_2_ fold change in the MPigut-IVM after the feed deprivation period. Wald test analysis using DESEQ2 package also indicated that potential opportunistic pathogens could be favored by the feed deprivation period such as *Solobacterium* [65]*, Sphaerochaeta* [66]*, Peptoniphilus* [67] and two Enterobacteriaceae family members, *Proteus* [68] and *Escherichia-Shigella* genera [69]. The effect of the feed deprivation period in the MPigut-IVM was markedly observed in 2 out of the 4 fermentations which harbored the higher percentage of Enterobacteriaceae after the stabilization period. Interestingly, the fermentations #6 and 7 which displayed the highest increase in Enterobacteriaceae and *Escherichia coli* were also those for which the redox potential was the most impacted by the feed deprivation stress. The reasons for this increase in redox potential after the feed deprivation stress in the MPigut-IVM remain unclear but certainly due to reducing availability of nutrients and thus reduced fermentative activity. In this study, we notice that the highest increases of the redox potential were not correlated with the highest increases of oxygen percentages in the bioreactors. Additionally, air contamination was avoided by the connection to the bioreactor of a gas bag filled with CO_2_ when the bioreactors was under pressure during feed deprivation. Redox potential reflects the reducing capacity of the gut environment which is influenced by several factors such as for instance diffusion of oxygen or release of reactive oxygen species or reactive nitrate species [70–72]. The relationship between intestinal redox potential, diet and intestinal microbiota of piglets is not elucidated yet. In a study from Xu et al. [73], early weaning was associated to a decrease in antioxidant capacities, increases in colon hydroxyl radicals and H_2_O_2_ and increase in *Escherichia coli* counts. *Escherichia* species already proved to be tolerant to oxidative stress contrary to other species from the gut microbiota which possess a high sensitivity [74]. We could speculate that individuals possessing more oxidative stress-resistant bacterial species would be more competitive towards Enterobacteriaceae and would thus be less impacted by an increase of redox potential*.* These hypotheses could explain the variable effects of the feed deprivation stress on the Enterobacteriaceae family inside our MPigut-IVM.

In our *in vitro* model, the feed deprivation stress led to a decrease in total SCFA concentration in the bioreactor medium after the end of 48 h stress period which was already observed in the cecum of suckling piglets after early exposure to antibiotics and could be referred to a dysbiotic state [75]. *In vivo*, modifications of SCFA profiles would have a direct impact on intestinal enterocytes but also on gut homeostasis [76]. In addition to SCFA, metabolomics analysis showed that numbers of bacterial metabolites were significantly impacted by *in vitro-*simulated feed deprivation period. Indeed, biogenic amines and amino acids were decreased by the feed deprivation stress suggesting a reduction in proteolytic and peptidolytic bacterial activity. Amino acid components such as alanine, valine, leucine and isoleucine, were however increased both in the bioreactor medium and the mucin beads during the recovery period indicating strong resilience of the microbiota. Since microbial metabolites are considered as key molecular intermediates between the microbiota and host cells, a perspective of our work would be to treat pig intestinal epithelial cells (e.g. IPEC-J2 or organoids) with bacterial metabolites produced in the MPigut-IVM, alone or in combination. These experiments could improve our understanding of the molecular links between weaning-induced dysbiosis and gut barrier dysfunction in piglets.

Our study also reported strong correlations between some bacterial families and the *in vitro* metabolome detected by NMR which was for the first time performed in *in vitro* models. These findings highlighted the association of populations such as Ruminococcaceae, Clostridiaceae, Prevotellaceae or Bacteroidiaceae with production of SCFAs [76] which confirmed that important functional groups are able to maintain their activity in the MPigut-IVM. Less known components such as polyamines, especially putrescine and cadaverine, were also detected in our samples and correlated with bacterial taxa. These molecules, absorbed by the host intestine, have a wide panel of biological functions like epigenetic regulations, stress resistance or cell proliferation [77] and could potentially have a role in the physiological consequences of the weaning period. Production and degradation of polyamines by the gut microbiota remain unclear but some bacterial taxa such as *Bifidobacterium* and *Escherichia coli* have been associated with putrescine production [78]. Interestingly, in our *in vitro* model, Bifidobacteriaceae and Enterobacteriaceae families were positively correlated with a higher concentration of cadaverine. Further investigations would be needed to understand the metabolic role of this bacterial communities during weaning associated feed deprivation stress. Our metabolome and correlation data however highlighted that the MPigut-IVM was able to mimic the very complex colonic environment up to deciphering the production of a rich panel of microbial metabolites.

*In vivo*, weaning transient anorexia could lead to a more competitive environment for nutrient degradation inside the piglet colon and, such as observed in our *in vitro* model, disruptions of microbiome and metabolome leading to intestinal dysbiosis and post weaning infections. The few days following low feed intake at weaning seem to be a critical period during which high microbial competition occurs and the most robust species such as opportunistic pathogens could take the advantage to colonize released ecological niches. Further research using mucin associated *in vitro* model such as our MPigut-IVM including other weaning stress factors such as the presence of pathogens or a dietary change should benefit to a better understanding of the etiology of intestinal dysbiosis in post-weaning piglets. Ultimately, the MPigut-IVM could be used to evaluate the impact of potential non-pharmalogical therapeutic alternatives in order to limit the use of antibiotics to treat post-weaning diarrhea in piglets.

## Conclusions

To conclude, our study reported the development of an innovative *in vitro* model of the piglet colon microbiota able to mimic gut luminal and mucus environments, while maintaining strict anaerobic conditions by the sole activity of the *in vitro* resident microbiota. We highlighted that weaning transient feed deprivation could lead to alterations of microbiota composition and metabolic profiles and might play a role in pathogen emergence. Despite the use of pooled fecal samples, some variability was conserved which will be of a great use for testing the effect of stressors, feed additives or pharmaceutical products in a more robust and relevant manner.

## Supplementary Information

**Additional file 1: Supplementary Table 1.** Composition of the nutritive medium simulating the ileal chyme of 28 d old piglets. **Supplementary Table 2.** Primers and probes used for quantitative experiments on the MPigut-IVM *in vitro* gut microbiota**. Supplementary Table 3.** Number of sequences generated by the Illumina MiSeq run. **Supplementary Table 4.** Statistical analysis of the principal phyla and families detected by Illumina sequencing in the bioreactor (A) and on the mucin beads (B) during the fermentation #6, 7, 8 and 9 which were subjected to a feed deprivation stress of 48 h (*n* = 4 for each time point).. Means associated with a different letter are significantly different. *P* adj: adjusted *P*-values (FDR method). **Supplementary Table 5.** Identification of metabolites in NMR spectra. The numbers are reported in representative spectra in supplemental figure 1. s: singulet. d: doublet. t: triplet. q: quintuplet; m: multiplet. *: indicate the peak used for quantification. **Supplementary Table 6.** Statistical analysis of metabolites detected by NMR metabolomics in the bioreactor and bead medium. Means associated with a different letter are significantly different. P adj: adjusted *P*-values (FDR method). **Supplementary Figure 1.** Identification of metabolites in a representative NMR spectrum**.** Peaks are identified with a number corresponding to the metabolites described in supplementary Table 5. The inset shows the aromatic region (vertically expanded). **Supplementary Figure 2.** Composition and metabolic activity of the microbiota from the fecal inoculi in all fermentation runs**:** relative abundance of SCFA measured by gas chromatography (A), quantification of bacterial groups using QPCR (*n* = 6) (B) and relative abundance of the principal bacterial phyla (C), bacterial families (D), archaeal families (E) and alpha diversity based on Shannon index and the number of observed OTUs (F) measured by 16S Illumina sequencing. “F” = “Fermentation”. **Supplementary Figure 3.** *In vitro* microbiota composition inside the bioreactor medium (A) and on the mucin beads (B) of the MPigut-IVM during control assays (fermentations #1, 2 and 3), as measured by QPCR. **Supplementary Figure 4.** Relative abundance of the main bacterial phyla in the bioreactor medium (A) and on the mucin beads (B) of the MPigut-IVM during control assays (fermentations #1, 2 and 3), as measured by 16S Illumina sequencing. **Supplementary Figure 5.** Mean relative abundance of the archaeal families in the bioreactor medium (A) and on the mucin beads (B) of the MPigut-IVM during control assays (fermentations #1, 2 and 3), as measured by 16S Illumina sequencing. **Supplementary Figure 6.** Percentages of variation calculated to estimate the best day for the end of the stabilisation phase using the relative abundances of gas, SCFA and the top 10 families of the MPigut-IVM. **Supplementary Figure 7.** Principal component analysis (PCoA) plot with Bray-Curtis dissimilarity on the bacterial communities between *in vivo* samples from liminal and mucosal proximal colon and *in vitro* fermentation samples from the bioreactor medium and the mucin beads at day 7 corresponding to the end of stabilization phase. **Supplementary Figure 8.** *In vitro* microbiota composition in the bioreactor medium (a, b and c) and on the mucin beads (d, e and f) of the MPigut-IVM during the simulation of 12 h (a and d), 24 h (b and e) et 48 h (c and f) food deprivation stresses, as measured by QPCR (*n* = 1 for each time point). **Supplementary Figure 9.** Relative abundance of the main bacteria phyla in the bioreactor medium (A) and on the mucin beads (B) in the MPigut-IVM during the fermentations #6, 7, 8 and 9 which were subjected to a food deprivation stress of 48 h. **Supplementary Figure 10.** Relative abundance of the main bacteria phyla (A) and families (B) in the bead medium of the MPigut-IVM during the fermentations #6, 7, 8 and 9 which were subjected to a food deprivation stress of 48 h. **Supplementary Figure 11.** Alpha diversity measures on bacterial OTUs in the bioreactor medium (A) and on the mucin beads (B) of the MPigut-IVM during the fermentations #6, 7, 8 and 9 which were subjected to a food deprivation stress of 48 h (*n* = 4). **Supplementary Figure 12.** Evolution of the redox potential inside the MPigut-IVM during the fermentations #6, 7, 8 and 9 which were subjected to a 48 h food deprivation stress. **Supplementary Figure 13.** Effect of a food deprivation stress of 48 h on the gas composition inside the MPigut-IVM. This figure displays mean relative abundance values of gases and their error bars collected during the fermentations #6, 7, 8 and 9 (*n* = 4 for each time point).

## Data Availability

The data generated and/or analysed during the current study are available in the BioProject database repository, https://www.ncbi.nlm.nih.gov/bioproject/615969.
