# Supplementary material for: Weaning-associated feed deprivation stress causes microbiota disruptions in a novel mucin-containing in vitro model of the piglet colon (MPigut-IVM)
Source: J Anim Sci Biotechnol. 2021 Jun 2;12:75. doi: 10.1186/s40104-021-00584-0 (PMC8170946; doi:10.1186/s40104-021-00584-0)
Supplement: Supplementary file 1 — Additional file 1: Supplementary Table 1. Composition of the nutritive medium simulating the ileal chyme of 28 d old piglets. Supplementary Table 2. Primers and probes used for quantitative experiments on the MPigut-IVM in vitro gut microbiota. Supplementary Table 3. Number of sequences generated by the Illumina MiSeq run. Supplementary Table 4. Statistical analysis of the principal phyla and families detected by Illumina sequencing in the bioreactor (A) and on the mucin beads (B) during the fermentation #6, 7, 8 and 9 which were subjected to a feed deprivation stress of 48 h (n = 4 for each time point).. Means associated with a different letter are significantly different. P adj: adjusted P-values (FDR method). Supplementary Table 5. Identification of metabolites in NMR spectra. The numbers are reported in representative spectra in supplemental figure 1. s: singulet. d: doublet. t: triplet. q: quintuplet; m: multiplet. *: indicate the peak used for quantification. Supplementary Table 6. Statistical analysis of metabolites detected by NMR metabolomics in the bioreactor and bead medium. Means associated with a different letter are significantly different. P adj: adjusted P-values (FDR method). Supplementary Figure 1. Identification of metabolites in a representative NMR spectrum. Peaks are identified with a number corresponding to the metabolites described in supplementary Table 5. The inset shows the aromatic region (vertically expanded). Supplementary Figure 2. Composition and metabolic activity of the microbiota from the fecal inoculi in all fermentation runs: relative abundance of SCFA measured by gas chromatography (A), quantification of bacterial groups using QPCR (n = 6) (B) and relative abundance of the principal bacterial phyla (C), bacterial families (D), archaeal families (E) and alpha diversity based on Shannon index and the number of observed OTUs (F) measured by 16S Illumina sequencing. “F” = “Fermentation”. Supplementary Figure 3. In vitro microbiota c [file 40104_2021_584_MOESM1_ESM.docx]

**Supplementary Table 1.** Composition of the nutritive medium simulating the ileal chyme of 28 days old piglets.

| Ingredients | g/L |
| --- | --- |
| Wheat starch | 5 |
| Pectin | 2 |
| Arabinogalactan | 2 |
| Cellulose | 2 |
| Soy protein isolate | 10 |
| Tryptone from casein | 4.5 |
| Mucin from porcine stomach type II | 2 |
| Whey powder | 5 |
| FeSO_4_, 7H_2_O | 0.005 |
| L-cysteine HCl monohydrate | 0.80 |
| Bile extract porcine | 0.40 |
| KH_2_PO_4_ | 0.5 |
| NaHCO_3_ | 1.5 |
| Hemin from porcine | 0.005 |
| NaCl | 4.5 |
| KCl | 4.5 |
| MgSO_4_ anhy. (120.37 g/mol) | 0.64 |
| CaCl_2_ 2H_2_O (147.02 g/mol) | 0.15 |
| MnCl_2_ 4H_2_O (197.91 g/mol) | 0.20 |

**Supplementary Table 2.** Primers and probes used for quantitative experiments on the MPigut-IVM in vitro gut microbiota**.**

| Target | Type | Sequences | References | Concentration | Annealing temperature |
| --- | --- | --- | --- | --- | --- |
| *Escherichia coli/ Shigella* group | Taqman | 5’-CAT GCC GCG TGT ATG AAG AA-3’  5’-CGG GTA ACG TCA ATG AGC AAA-3’  (6-FAM)-5’-TAT TAA CTT TAC TCC CTT CCT CCC CGC TGA A-3’(TAMRA) | 31 | 300 nM  300 nM  100 nM | 61°C |
| Total Bacteria | SYBRGREEN | 5’-ACT CCT ACG GGA GGC AG-3’  5’-GTA TTA CCG CGG CTG CTG-3’ | 32 | 500 nM  500 nM | 61°C |
| Firmicutes | SYBRGREEN | 5’-TGA AAC TYA AAG GAA TTG ACG-3’  5’-ACC ATG CAC CAC CTG TC-3’ | 33 | 500 nM  500 nM | 60°C |
| Bacteroidetes | SYBRGREEN | 5’-CRA ACA GGA TTA GAT ACC CT-3’  5’-GGT AAG GTT CCT CGC GTA T-3’ | 33 | 500 nM  500 nM | 60°C |
| *Prevotella* | SYBRGREEN | 5’-GGT TCT GAG AGG AAG GTC CCC-3’  5’-TCC TGC ACG CTA CTT GGC TG-3’ | 34 | 500 nM  500 nM | 60°C |
| *Clostridium* cluster IV | Taqman | 5‘-GCA CAA GCA GTG GAG T-3’  5’-CTT CCT CCG TTT TGT CAA-3’  (6-FAM)-AGG GTT GCG CTC GTT-(BHQ-1) | 35 | 500 nM  500 nM  300 nM | 55°C |
| *Clostridium* cluster XIVa | Taqman | 5’-GCA GTG GGG AAT ATT GCA-3’  5’-CTT TGA GTT TCA TTC TTG CGA A-3’  (6-FAM)-AAATGACGGTACCTGACTAA-(BHQ-1) | 35 | 500 nM  500 nM  300 nM | 56°C |
| Methanogenic archaea | SYBRGREEN | 5’-GAG GAA GGA GTG GAC GAC GGTA-3’  5’-ACG GGC GGT GTG TGC AAG-3’ | 36 | 500 nM  500 nM | 61°C |

**Supplementary Table 3.** Number of sequences generated by the Illumina MiSeq run.

|  | Number of sequences | |
| --- | --- | --- |
|  | Bioreactor medium | Mucin beads |
| Bacteria | 35,624 ± 7,371 per sample | 27,718 ± 11,550 per sample |
| Archaea | 16,574 ± 7,499 per sample | 6,686 ± 4,762 per sample |
|  | Total | |
| Bacteria | 4,463,192 | |
| Archaea | 3,239,771 | |

**Supplementary Table 4:** Statistical analysis of the principal phyla and families detected by Illumina sequencing in the bioreactor (A) and on the mucin beads (B) during the fermentation #6, 7, 8 and 9 which were subjected to a feed deprivation stress of 48h (n = 4 for each time point).. Means associated with a different letter are significantly different. P adj : adjusted p-values (FDR method).

**A**

|  | *p values* | | *Time points (days)* | | | | | |
| --- | --- | --- | --- | --- | --- | --- | --- | --- |
| Bacterial groups | **pval** | **padj** | **7** | **9** | **9.5** | **10** | **11** | **15** |
| *Actinobacteria* | 0.004 | 0.011 | ab | a | ab | b | b | b |
| *Bacteroidetes* | 0.006 | 0.011 | b | ab | a | ab | ab | b |
| *Firmicutes* | 0.011 | 0.011 | ab | ab | b | ab | ab | a |
| *Proteobacteria* | 0.011 | 0.011 | a | ab | ab | ab | ab | b |
| *Atopobiaceae* | 0 | 0 | a | ab | c | bc | ab | a |
| *Coriobacteriaceae* | 0.001 | 0.002 | b | a | ab | b | b | b |
| *Lachnospiraceae* | 0.002 | 0.002 | b | ab | a | ab | b | ab |
| *Erysipelotrichaceae* | 0 | 0 | a | bc | d | cd | bc | b |
| *Prevotellaceae* | 0 | 0 | c | a | b | c | c | c |
| *Veillonellaceae* | 0 | 0.001 | a | a | b | ab | ab | a |
| *Enterococaceae* | 0.001 | 0.002 | ab | a | b | ab | b | b |
| *Bacteroidiaceae* | 0 | 0 | b | b | ab | a | a | b |
| *Desulfovibrionaceae* | 0.023 | 0.046 | a | a | ab | ab | ab | b |
| *Ruminococcaceae* | 0 | 0 | b | b | ab | a | a | b |

**B**

|  | *p values* | | *Time points (days)* | | | |
| --- | --- | --- | --- | --- | --- | --- |
| Bacterial groups | **pval** | **padj** | **7** | **9** | **11** | **15** |
| *Actinobacteria* | 0.222 | 0.359 | a | a | a | a |
| *Bacteroidetes* | 0.359 | 0.359 | a | a | a | a |
| *Firmicutes* | 0.332 | 0.359 | a | a | a | a |
| *Proteobacteria* | 0.121 | 0.359 | a | a | a | a |
| *Bifidobacteriaceae* | 0.009 | 0.01 | b | b | ab | a |
| *Peptostreptococcaceae* | 0 | 0 | c | c | b | a |
| *Eubacteriaceae* | 0 | 0 | b | b | a | a |
| *Clostridiaceae.1* | 0 | 0 | c | c | b | a |
| *Spirochaetaceae* | 0 | 0 | b | b | a | a |
| *Ruminococcaceae* | 0 | 0 | b | b | a | a |
| *Veillonellaceae* | 0.009 | 0.01 | ab | b | b | a |
| *Succinivibrionaceae* | 0.001 | 0.001 | bc | c | ab | a |

**Supplementary Table 5:** Identification of metabolites in NMR spectra. The numbers are reported in representative spectra in supplemental figure 1. s: singulet. d: doublet. t: triplet. q: quintuplet; m: multiplet. *: indicate the peak used for quantification.

|  | Metabolite | δ^1^H (ppm) |
| --- | --- | --- |
| 1 | 2-methylbutyrate | 0.86 (t). 1.39* (m) |
| 2 | Valerate | 0.89 (t). 1.31* (m). 1.53 (m). 2.19 (t) |
| 3 | Butyrate | 0.90* (t). 1.56 (m). 2.16 (t) |
| 4 | Isovalerate | 0.91 (d). 1.96 (m). 2.06* (d) |
| 5 | Isoleucine | 0.94 (t). 1.01* (d) |
| 6 | Leucine | 0.97* (t) |
| 7 | Valine | 1.00* (d). 1.05 (d). 3.62 (d) |
| 8 | Propionate | 1.06* (t). 2.19 (m) |
| 9 | Isobutyrate | 1.07 (d). 2.40* (m) |
| 10 | Ethanol | 1.19* (t). 3.66 (m) |
| 11 | Alanine | 1.48* (d). 3.79 (m) |
| 12 | 5-aminovalerate | 1.65 (m). 2.24* (t). 2.33 (t). 3.02 (t) |
| 13 | Cadaverine | 1.73* (m). 3.02 (t) |
| 14 | Putrescine | 1.78* (m). 3.05 (t) |
| 15 | Acetate | 1.92* (s) |
| 16 | p-cresol | 2.26 (s). 6.84* (d). 7.15 (d) |
| 17 | 3-(4-hydroxyphenyl)propionate | 2.45* (t). 2.82 (t). 6.86 (d). 7.18 (d) |
| 18 | 3-phenylpropionate | 2.50* (t). 2.89 (t). 7.27 (t). 7.32 (d). 7.37 (t) |
| 19 | Methylamine | 2.60* (s) |
| 20 | Trimethylamine | 2.88* (s) |
| 21 | Tyramine | 2.94 (t). 3.24 (t). 6.92* (d). 7.23 (d) |
| 22 | Methanol | 3.36* (s) |
| 23 | 2-(4-hydroxyphenyl)acetate | 3.45* (s). 6.87 (d). 7.17 (d) |
| 24 | Phenylacetate | 3.54* (s). 7.31 (t). 7.39 (t) |
| 25 | Glycine | 3.57* (s) |
| 26 | Formate | 8.46* (s) |

**Supplementary Table 6:** Statistical analysis of metabolites detected by NMR metabolomics in the bioreactor and bead medium. Means associated with a different letter are significantly different. P adj : adjusted p-values (FDR method).

|  | **Bioreactor medium** | | | | | | | | **Bead medium** | | | | | |
| --- | --- | --- | --- | --- | --- | --- | --- | --- | --- | --- | --- | --- | --- | --- |
|  | *P values* | | *Time points* | | | | | | *P values* | | *Time points* | | | |
|  | **pval** | **padj** | **7** | **9** | **9.5** | **10** | **11** | **15** | **pval** | **padj** | **7** | **9** | **11** | **15** |
| formate | 0.038 | 0.055 | a | a | a | a | a | a | 0.287 | 0.355 | a | a | a | a |
| tyramine | 0.000 | 0.000 | a | a | b | a | a | ab | 0.020 | 0.052 | ab | a | ab | b |
| p_cresol | 0.334 | 0.362 | a | a | a | a | a | a | 0.929 | 0.929 | a | a | a | a |
| glycine | 0.000 | 0.000 | ab | a | c | b | ab | ab | 0.083 | 0.160 | a | a | a | a |
| phenylacetate | 0.701 | 0.701 | a | a | a | a | a | a | 0.018 | 0.051 | a | b | ab | ab |
| 2_4_hydroxyphenylacetate | 0.021 | 0.034 | ab | a | ab | b | ab | ab | 0.077 | 0.160 | a | a | a | a |
| methanol | 0.000 | 0.000 | b | a | ab | b | b | b | 0.156 | 0.233 | a | a | a | a |
| trimethylamine | 0.001 | 0.002 | b | a | a | ab | ab | b | 0.000 | 0.001 | b | a | ab | b |
| methylamine | 0.070 | 0.091 | a | a | a | a | a | a | 0.003 | 0.023 | ab | b | a | a |
| 3_phenylpropionate | 0.053 | 0.072 | a | a | a | a | a | a | 0.351 | 0.415 | a | a | a | a |
| 3_4_hydroxyphenylpropionate | 0.000 | 0.000 | ac | a | a | bc | c | ab | 0.086 | 0.160 | a | a | a | a |
| isobutyrate | 0.022 | 0.034 | ab | a | ab | b | ab | b | 0.161 | 0.233 | a | a | a | a |
| 5_aminovalerate | 0.000 | 0.000 | b | a | ab | b | b | b | 0.059 | 0.139 | a | a | a | a |
| isovalerate | 0.000 | 0.000 | ab | bc | a | c | c | bc | 0.010 | 0.041 | ab | a | ab | b |
| acetate | 0.000 | 0.000 | bc | c | a | a | ab | ac | 0.225 | 0.308 | a | a | a | a |
| putrescine | 0.084 | 0.095 | a | a | a | a | a | a | 0.119 | 0.194 | a | a | a | a |
| cadaverine | 0.081 | 0.095 | a | a | a | a | a | a | 0.092 | 0.160 | a | a | a | a |
| alanine | 0.008 | 0.013 | b | a | b | ab | ab | ab | 0.006 | 0.040 | ab | a | b | b |
| 2_methylbutyrate | 0.000 | 0.000 | b | c | a | bc | bc | bc | 0.008 | 0.040 | ab | b | a | a |
| valerate | 0.004 | 0.008 | a | b | ab | ab | a | a | 0.015 | 0.049 | ab | b | ab | a |
| ethanol | 0.006 | 0.011 | ab | a | ab | b | ab | b | 0.516 | 0.559 | a | a | a | a |
| propionate | 0.074 | 0.091 | a | a | a | a | a | a | 0.886 | 0.922 | a | a | a | a |
| isoleucine | 0.000 | 0.000 | a | a | b | a | a | a | 0.391 | 0.442 | a | a | a | a |
| valine | 0.000 | 0.000 | bc | a | c | ab | ab | ab | 0.011 | 0.041 | b | a | ab | ab |
| leucine | 0.000 | 0.000 | b | a | c | ab | ab | ab | 0.000 | 0.005 | b | a | b | b |
| butyrate | 0.405 | 0.422 | a | a | a | a | a | a | 0.266 | 0.346 | a | a | a | a |

**Supplementary Figure 1**: Identification of metabolites in a representative NMR spectrum**.** Peaks are identified with a number corresponding to the metabolites described in supplementary Table 5. The inset shows the aromatic region (vertically expanded).


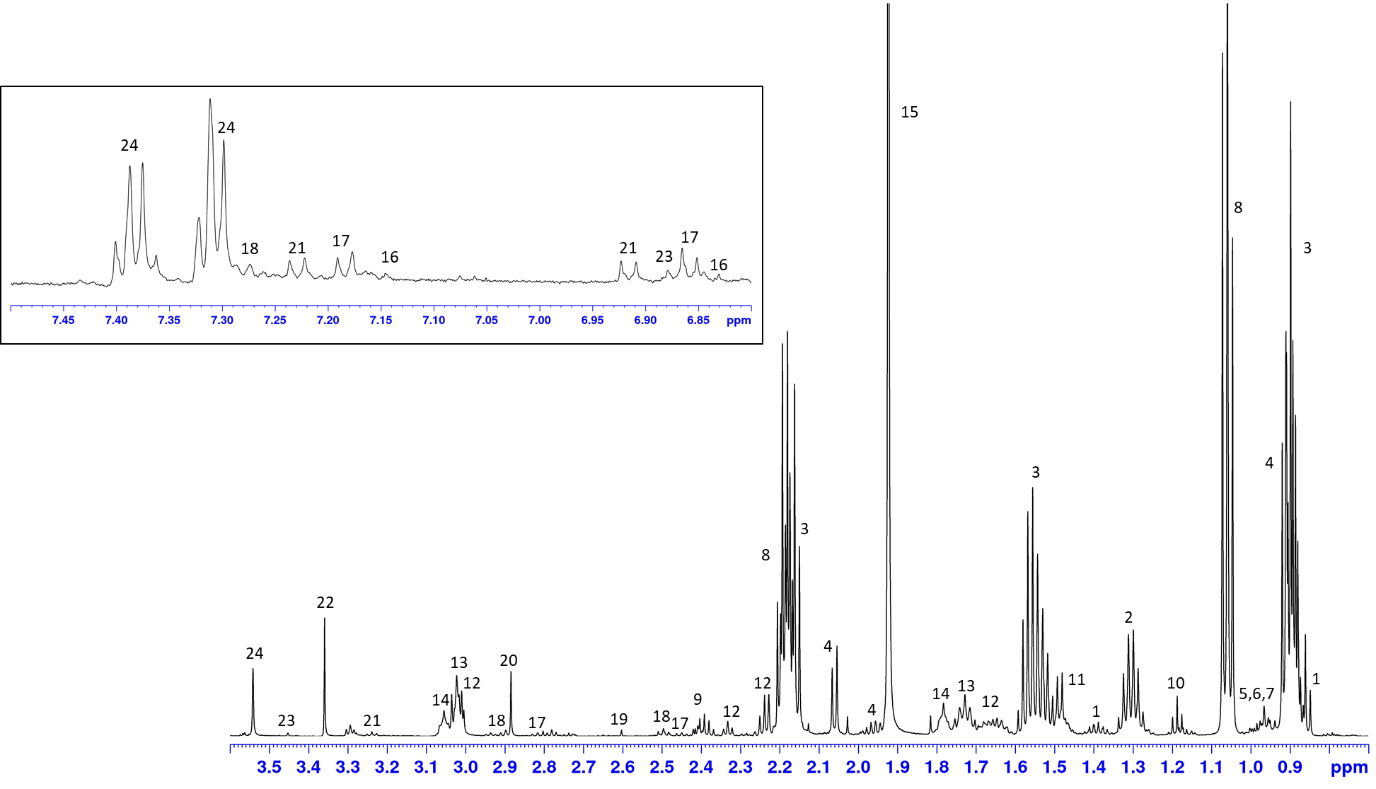


**Supplementary Figure 2.** Composition and metabolic activity of the microbiota from the fecal inoculi in all fermentation runs**:** relative abundance of SCFA measured by gas chromatography (A), quantification of bacterial groups using QPCR (n = 6) (B) and relative abundance of the principal bacterial phyla (C), bacterial families (D), archaeal families (E) and alpha diversity based on Shannon index and the number of observed OTUs (F) measured by 16S Illumina sequencing. “F” = “Fermentation”.


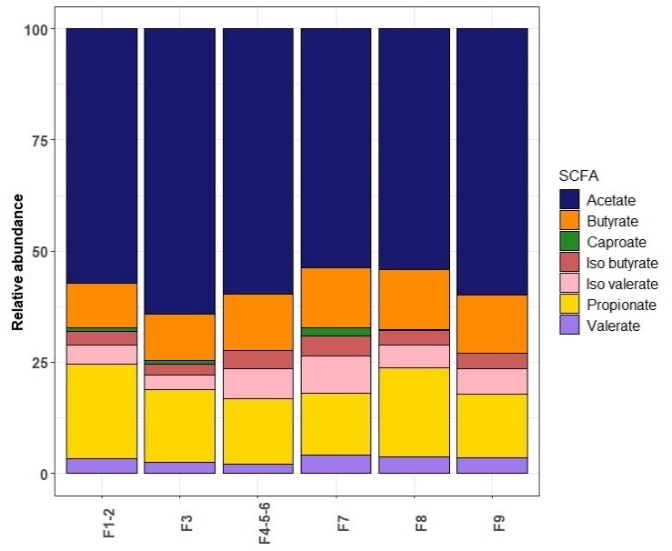


B

A


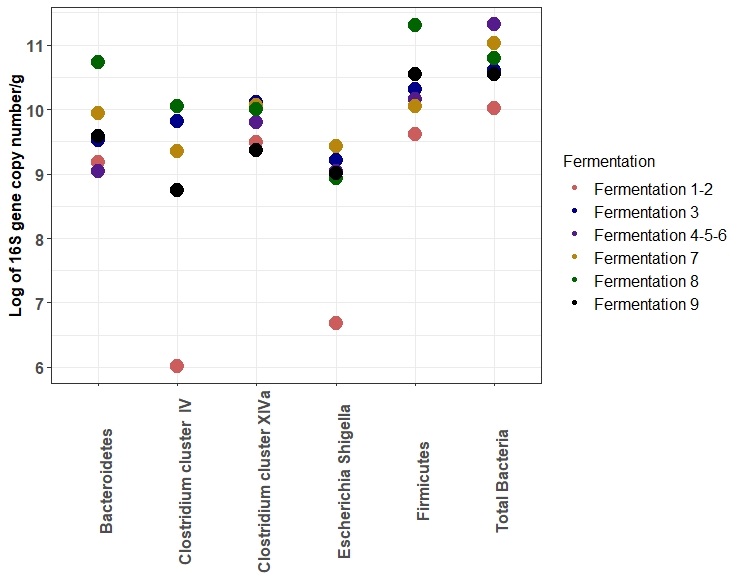


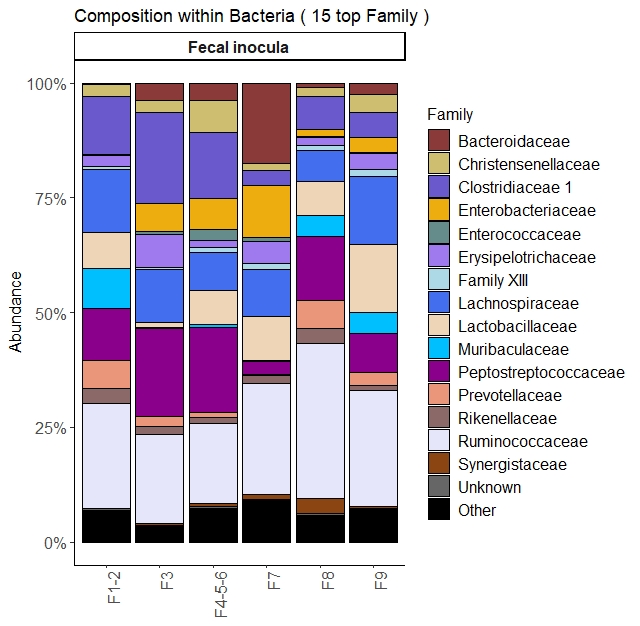

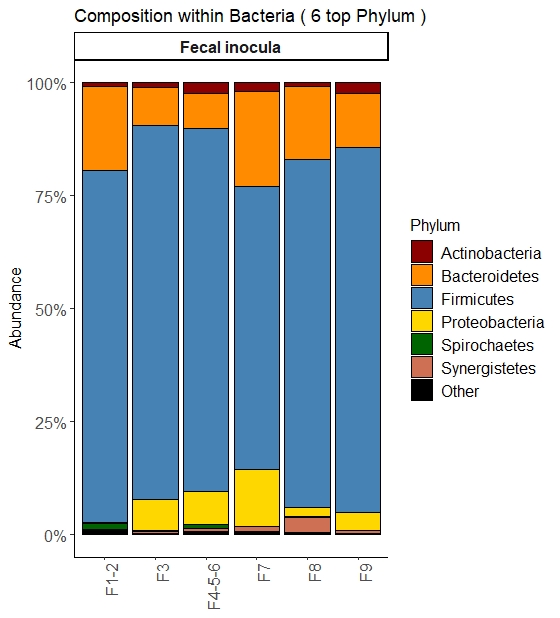


C

D


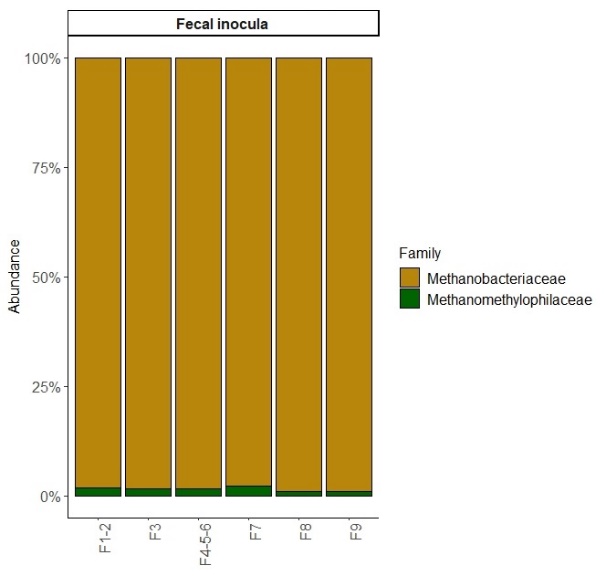

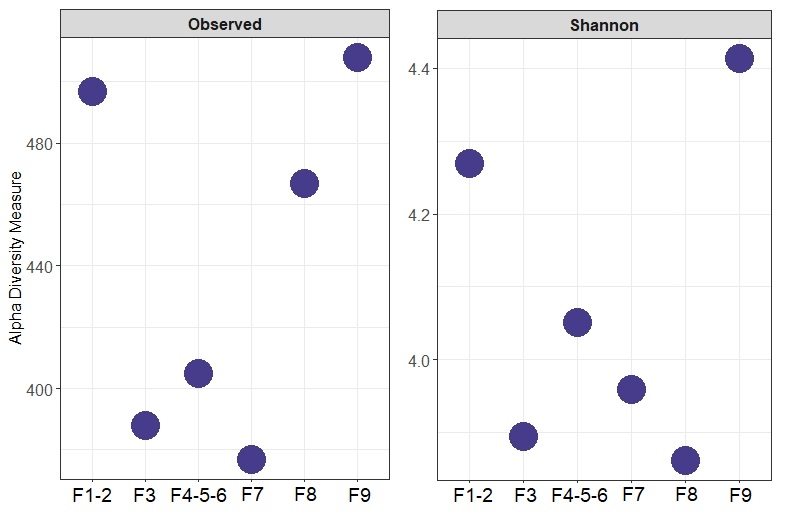


E

F

**Supplementary Figure 3.** *In vitro* microbiota composition inside the bioreactor medium (A) and on the mucin beads (B) of the MPigut-IVM during control assays (fermentations #1, 2 and 3), as measured by QPCR.

**A**


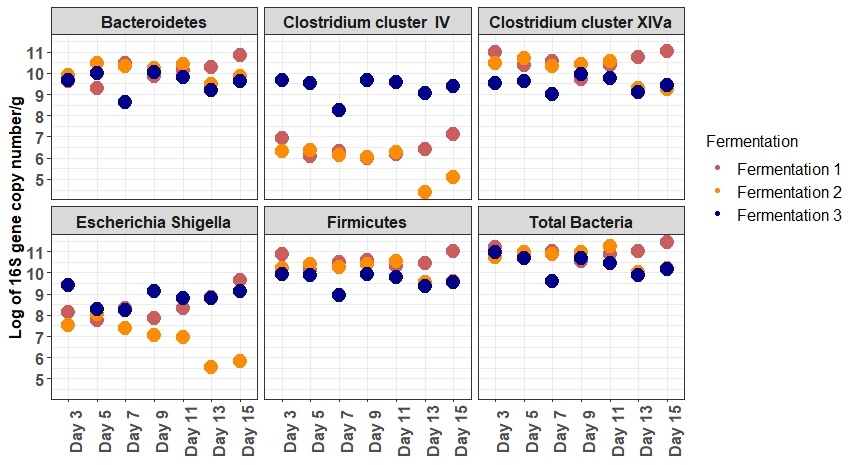


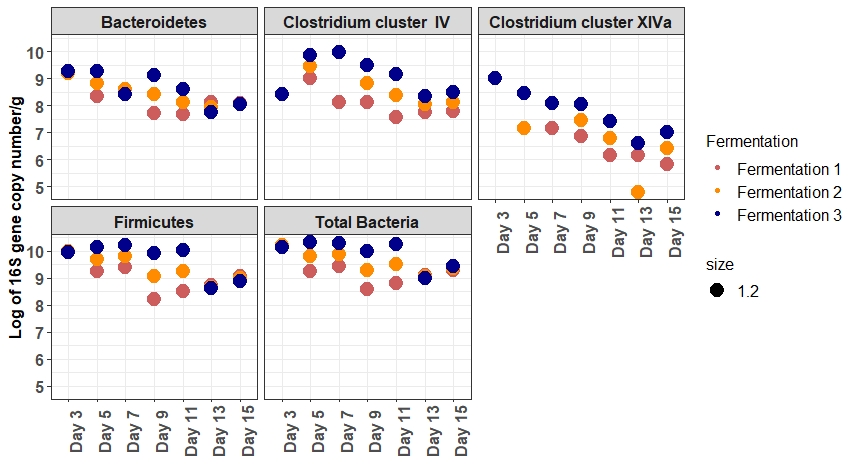


**B**

**Supplementary Figure 4.** Relative abundance of the main bacterial phyla in the bioreactor medium (A) and on the mucin beads (B) of the MPigut-IVM during control assays (fermentations #1, 2 and 3), as measured by 16S Illumina sequencing.

**A**

**
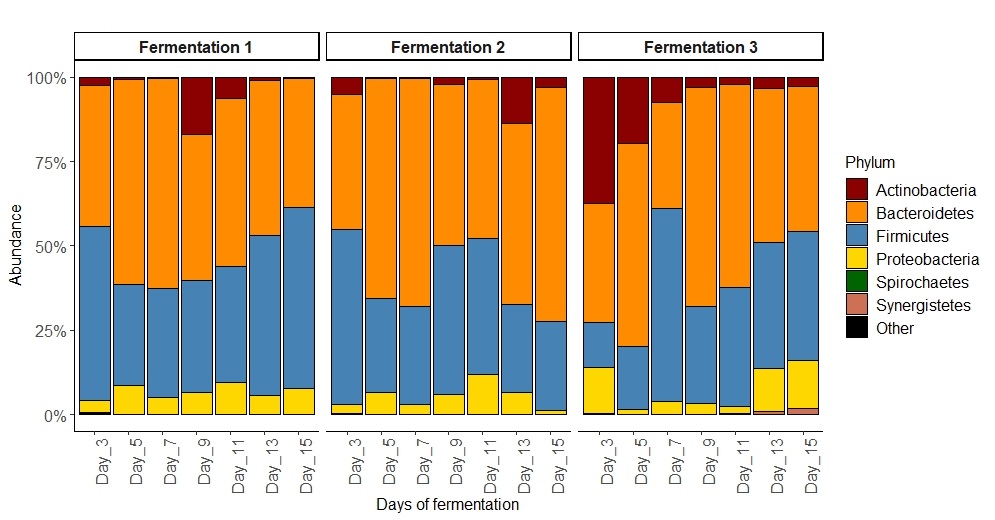
**


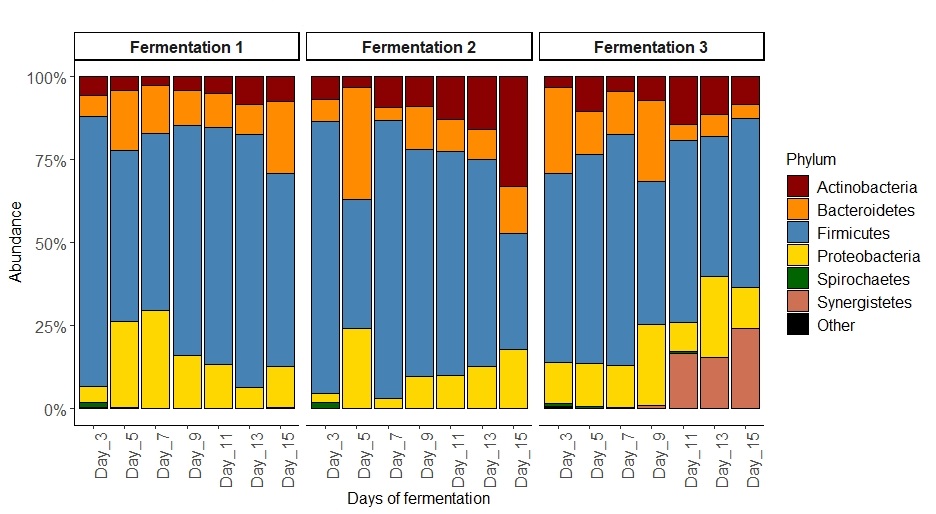


**B**

**Supplementary Figure 5.** Mean relative abundance of the archaeal families in the bioreactor medium (A) and on the mucin beads (B) of the MPigut-IVM during control assays (fermentations #1, 2 and 3), as measured by 16S Illumina sequencing.


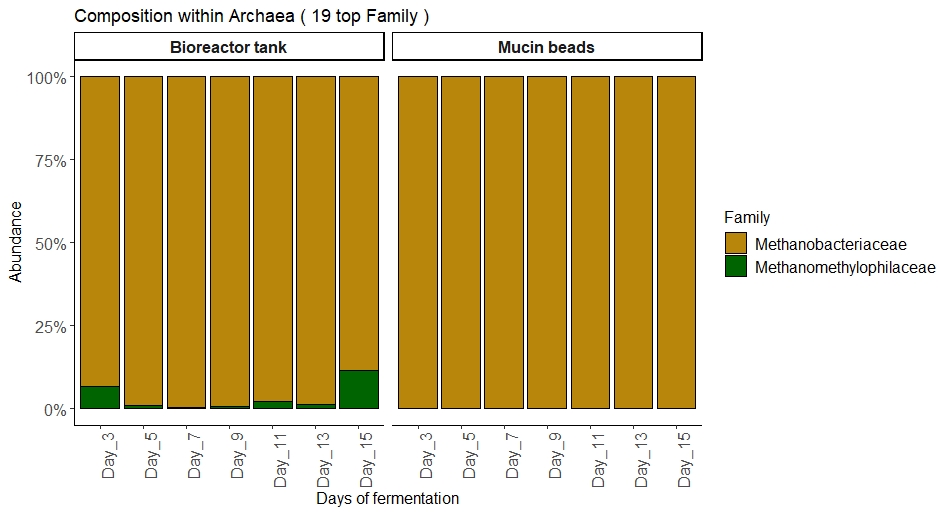


**Supplementary Figure 6.** Percentages of variation calculated to estimate the best day for the end of the stabilisation phase using the relative abundances of gas, SCFA and the top 10 families of the MPigut-IVM.


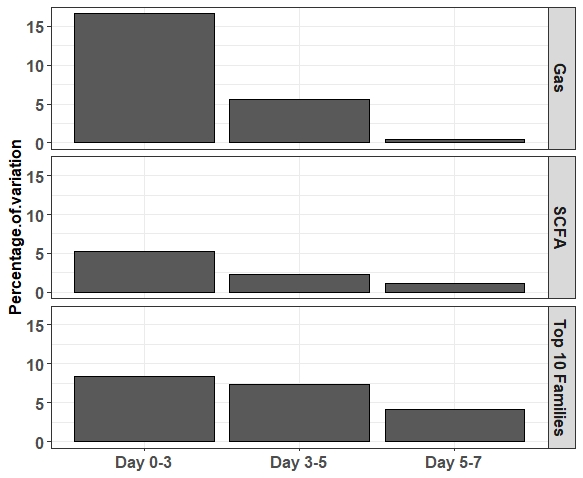


**Supplementary Figure 7.** Principal component analysis (PCoA) plot with Bray-Curtis dissimilarity on the bacterial communities between *in vivo* samples from liminal and mucosal proximal colon and *in vitro* fermentation samples from the bioreactor medium and the mucin beads at day 7 corresponding to the end of stabilization phase.


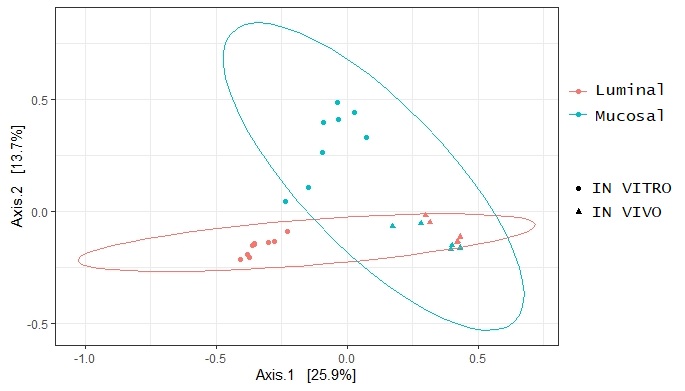


**Supplementary Figure 8.** *In vitro* microbiota composition in the bioreactor medium (a, b and c) and on the mucin beads (d, e and f) of the MPigut-IVM during the simulation of 12h (a and d), 24h (b and e) et 48h (c and f) food deprivation stresses, as measured by QPCR (n = 1 for each time point).


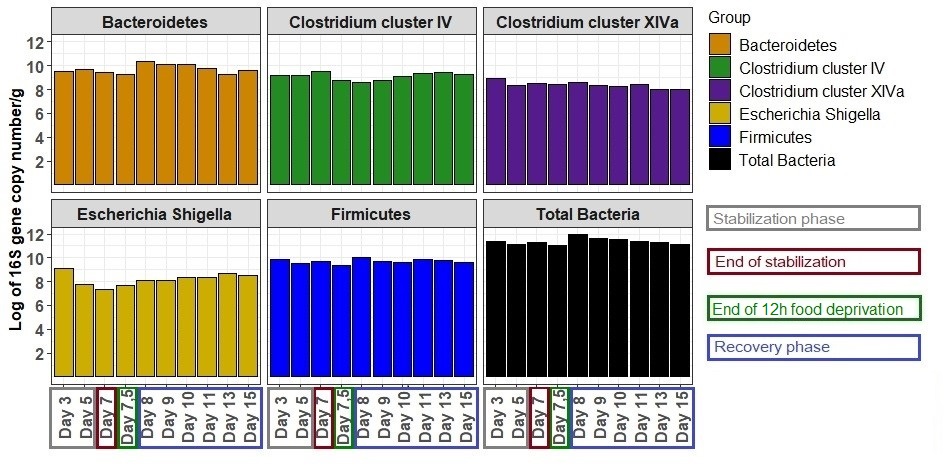


**a**


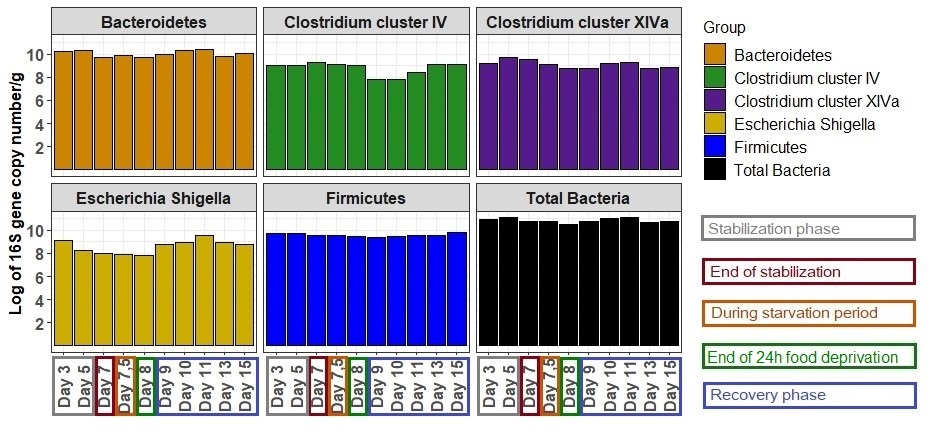


**b**


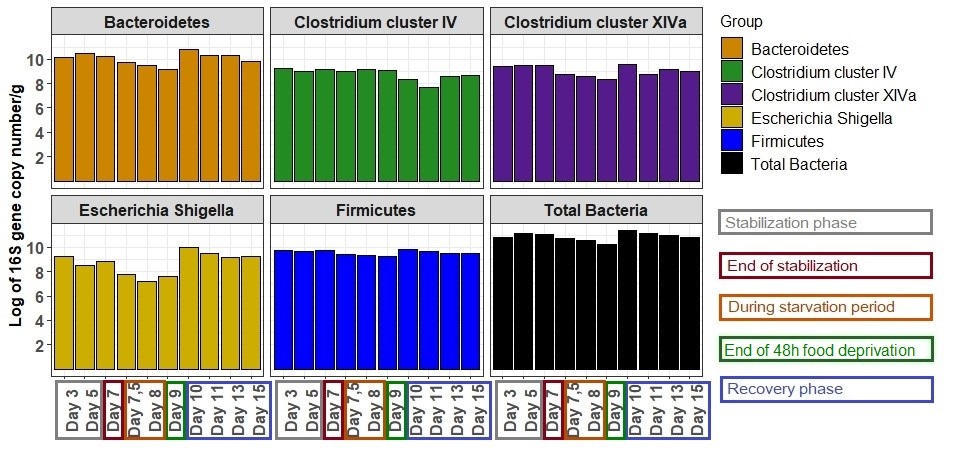


**c**


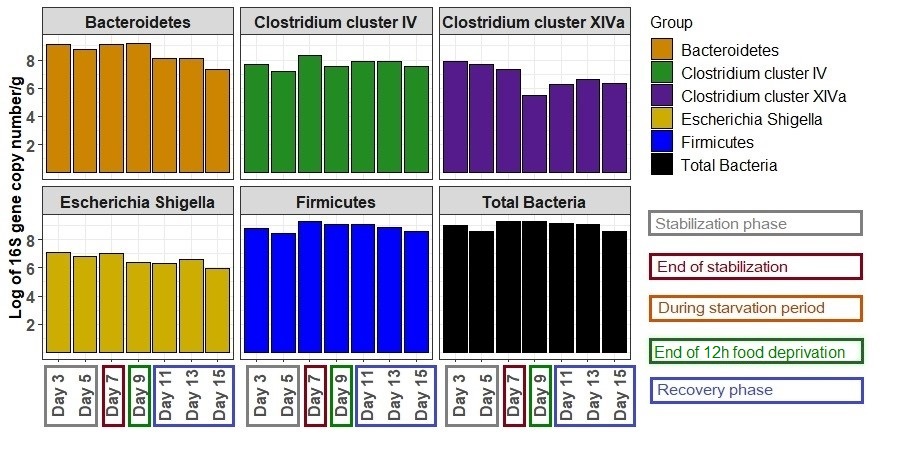


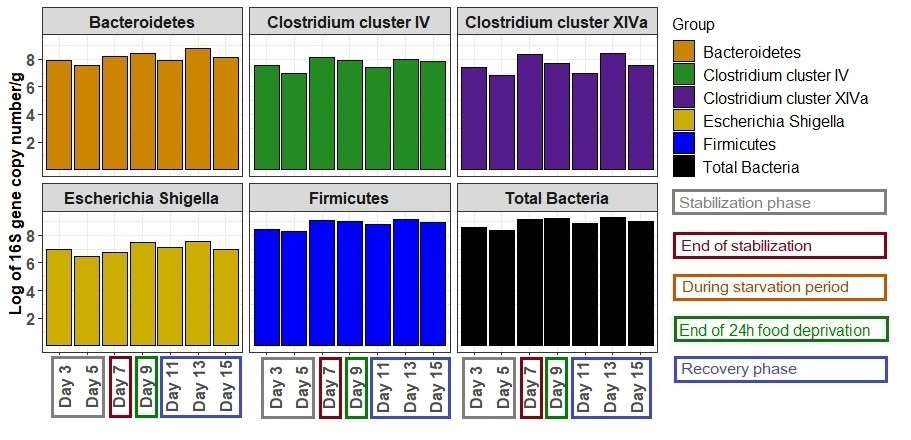


**e**

**d**


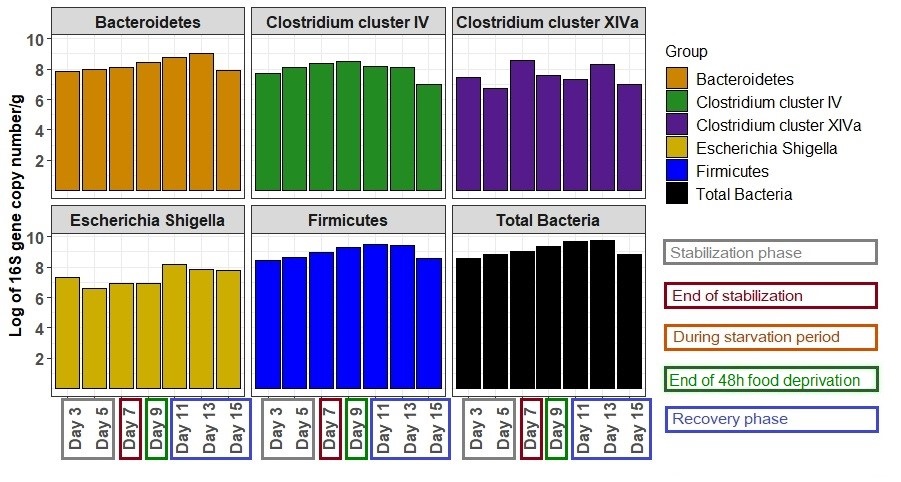


**f**

**Supplementary Figure 9.** Relative abundance of the main bacteria phyla in the bioreactor medium (A) and on the mucin beads (B) in the MPigut-IVM during the fermentations #6, 7, 8 and 9 which were subjected to a food deprivation stress of 48h.


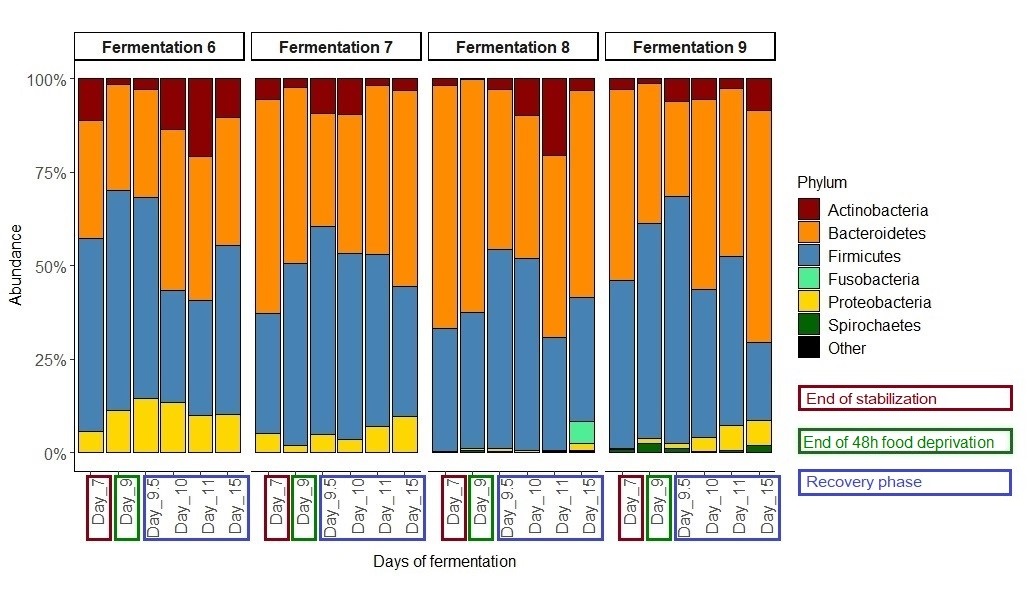


**A**


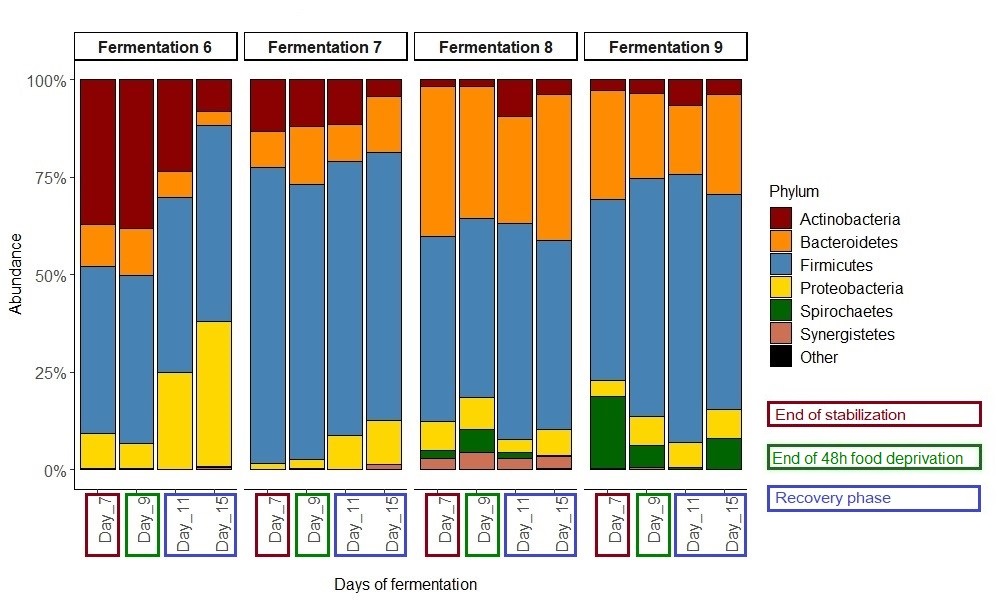


**B**

**Supplementary Figure 10.** Relative abundance of the main bacteria phyla (A) and families (B) in the bead medium of the MPigut-IVM during the fermentations #6, 7, 8 and 9 which were subjected to a food deprivation stress of 48h.


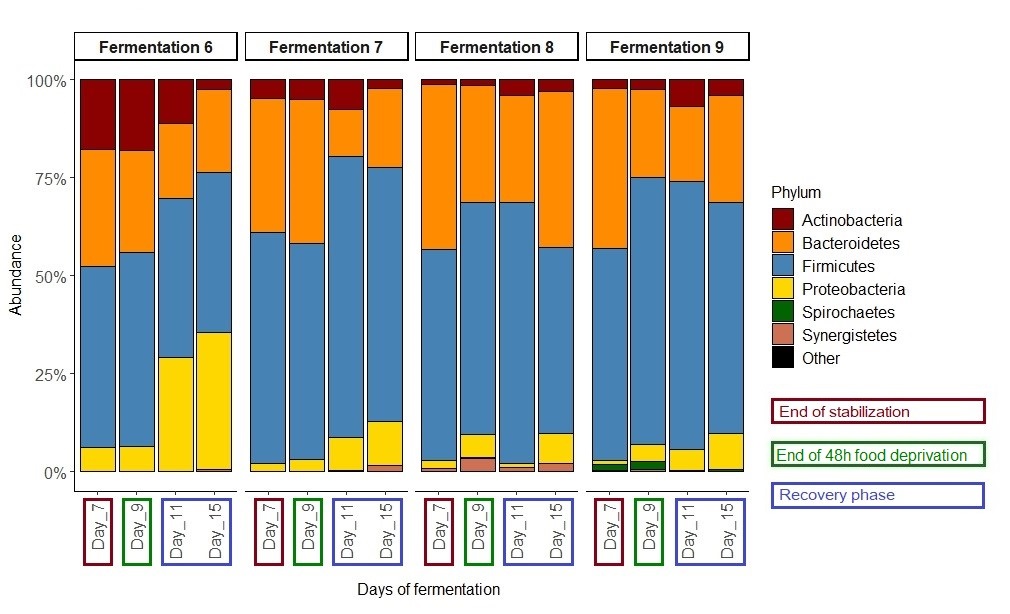


**A**


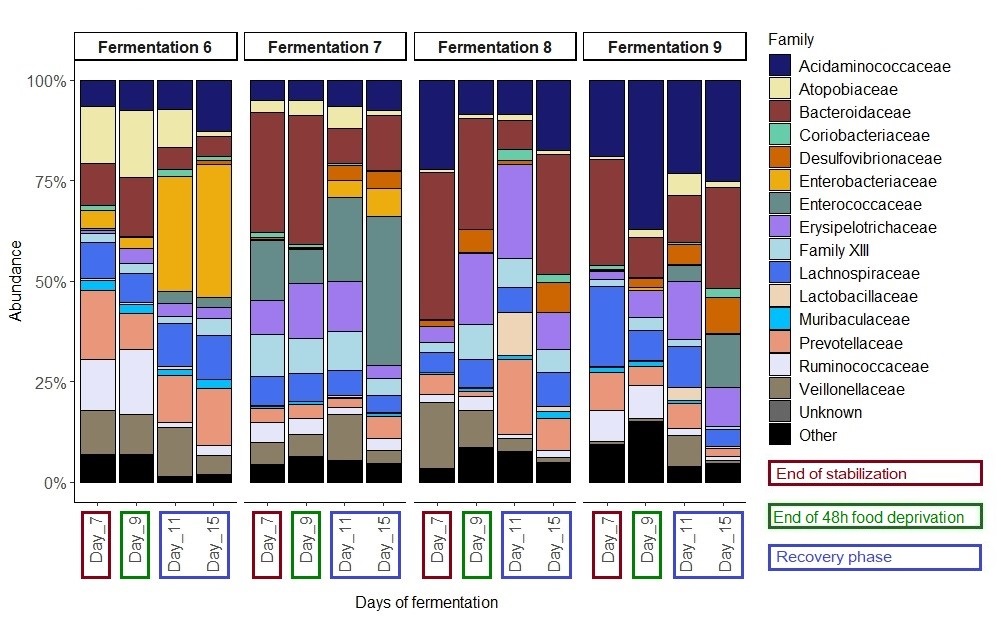


**B**

**Supplementary Figure 11.** Alpha diversity measures on bacterial OTUs in the bioreactor medium (A) and on the mucin beads (B) of the MPigut-IVM during the fermentations #6, 7, 8 and 9 which were subjected to a food deprivation stress of 48h (n = 4).


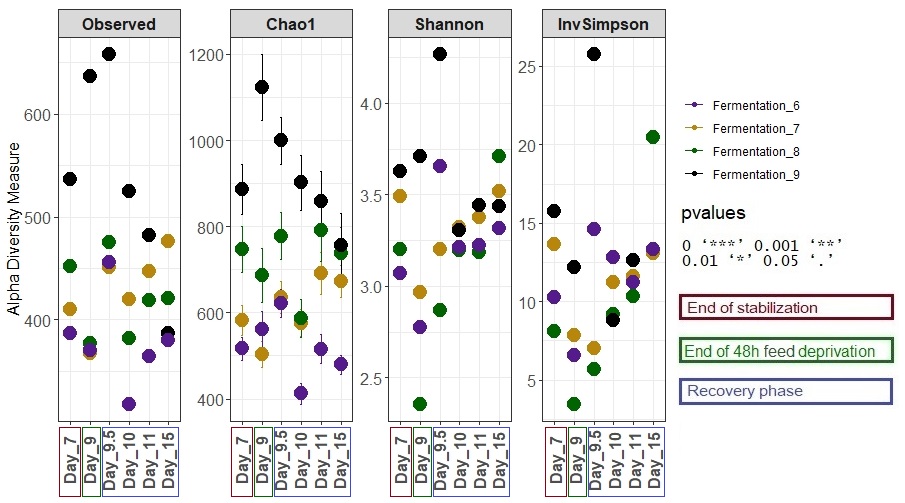


**A**


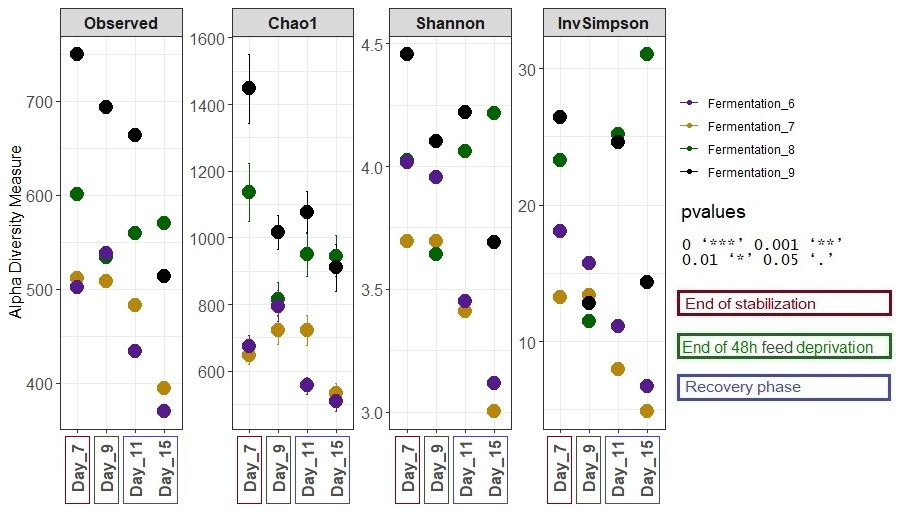


**B**

**Supplementary Figure 12.** Evolution of the redox potential inside the MPigut-IVM during the fermentations #6, 7, 8 and 9 which were subjected to a 48h food deprivation stress.


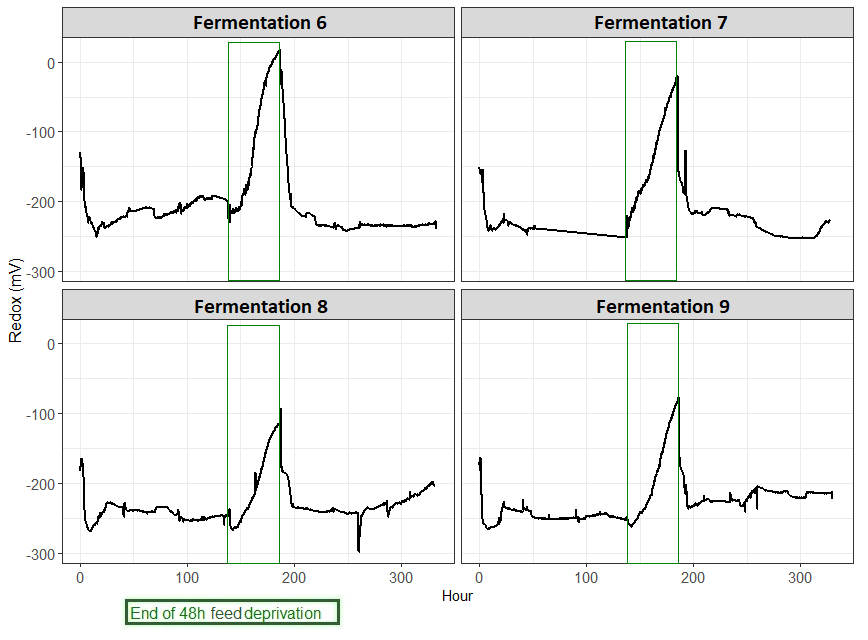


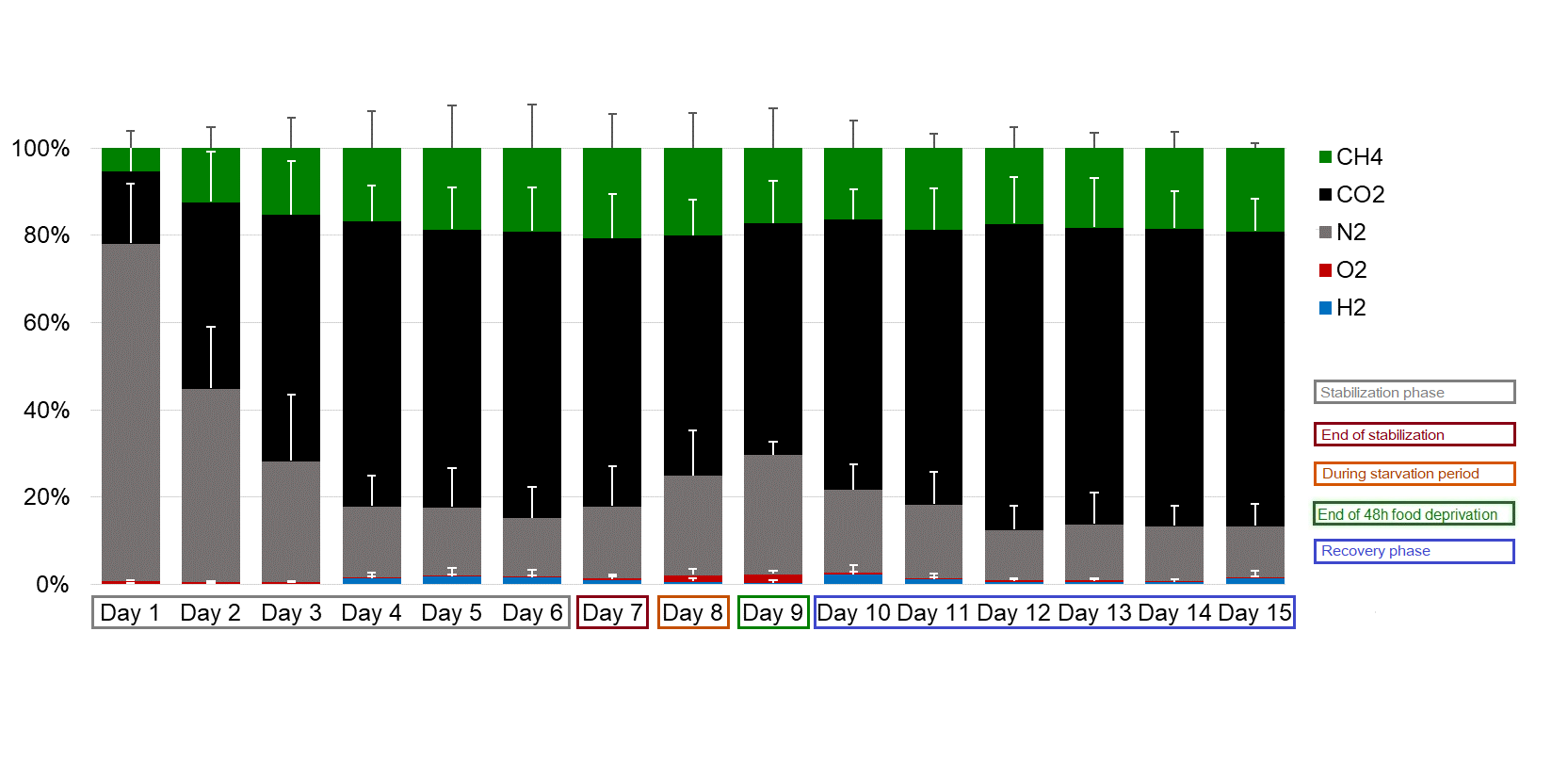
 **Supplementary Figure 13. Effect of a food deprivation stress of 48h on the gas composition inside the MPigut-IVM.** This figure displays mean relative abundance values of gases and their error bars collected during the fermentations #6, 7, 8 and 9 (n = 4 for each time point)
